# Oral Delivery of miRNA With Lipidic Aminoglycoside Derivatives in the Breastfed Rat

**DOI:** 10.3389/fphys.2019.01037

**Published:** 2019-08-13

**Authors:** Diane Beuzelin, Bruno Pitard, Bertrand Kaeffer

**Affiliations:** ^1^UMR 1280, NUN, Institut National de la Recherche Agronomique, Nantes, France; ^2^Centre de Recherche en Cancérologie et Immunologie Nantes Angers (CRCINA), Institut National de la Santé et de la Recherche Médicale (INSERM), Université d’Angers, Université de Nantes, Nantes, France

**Keywords:** miRNA, circadian clock, stomach, nanopackaging, chromatin immune-precipitation, q-PCR real-time

## Abstract

**Context:**

Specific targeting of endogenous miRNAs which are involved in epigenetics, may help understanding homeostasis with therapeutic benefits. We use new biologically inspired vehicles consisting of lipoaminoglycosides to deliver *in vivo* mir-320-3p, a known human breast milk exosomal miRNA, or its antagomiR.

**Materials and Methods:**

Four lipoaminoglycosides were screened for cytotoxicity and their biophysical properties. 1-h breast-restricted rats received single-oral treatment of either the lipoaminoglycoside Dioleyl-Succinyl Paromomycin (DOSP) complexed with miRNA or antagomiR, or of control medium at the light on (ZeitGeber Time: ZT-0H) or off (ZT-12H). Glycemia, triglycerides, cholesterol, free-fatty acid were assayed at 0, 4, 8, and 12 h post-treatment. In the stomach, small intestine, liver, plasma, adipose tissue, plexus choroid, and cortex, relevant miRNA with precursors and mRNA (polr3d, hspb6, c-myc, stat1, clock, bmal1, per1, npas2, sirt1-6, and cyclinD1) were quantified by q-PCR. Expression of POLR3D and HSPB6 proteins were analyzed in stomach and liver by Western blot. Immunoprecipitations with anti-AGO1 and 2 were performed on nuclear and cytoplasmic fractions of gastric cells along with detection of miRNA-320-3p in nucleoli. Chromatin ImmunoPrecipitation with anti-Trimethyl-histone-3-Lys-4 and Lys-27 detecting the polr3d promoter and miR-320-3p, were performed for all groups.

**Results:**

Selected DOSP (diameter: 80–200 nm) did not alter gastric extracellular vesicle secretion a few hours after intake. The miR-320-3p was mainly found in gastric or small intestinal cells, reaching the blood and liver in low amount. We have found significant up-regulation of polr3d mRNA (ANOVA, *p* < 0.0001) at ZT-20H for the miR-320-3p-supplemented group and a higher expression of POLR3D for antagomiR group (ANOVA, *p* < 0.05). We had a low accumulation of miR-320-3p at ZT-20H in nucleoli, without stat1 evolution. Delivering a high amount of miRNA or antagomiR disrupts RNA-Induced Silencing Complexes in cytoplasm triggering some transfer of extracellular molecules into nuclei with alteration of immune complexes on the polr3d promoter (with a higher amount found in the K4 histone-3-me3 immune complexes at ZT-20H).

**Conclusion:**

Extracellular miRNAs embedded in DOSP have a rapid impact on RNAi and on nuclear chromatin complexes depending on the daily rhythm. An integrative view of the impact of extracellular miRNA on physiology will improve assaying epigenetic manipulations following nutritional stress.

## Introduction

Non-coding RiboNucleic Acid (small or large nc-RNA) are involved in epigenetic regulation directly silencing chromatin at specific loci by base pairing to nascent transcripts (Cech and Steitz, 2014). Among these ncRNAs, miRNAs are proposed to play a significant role in epigenetic regulation opening new avenues for therapeutic purposes (Sethupathy, 2016) or for assaying exogenous non-coding RNA acquisition on the physiology of host and putative consequences across generations (Sela et al., 2014; Junien et al., 2016). Milk contains a high amount of miRNA which has been proposed for transfer between mother and child for immune regulation (Kosaka et al., 2010), for priming the immune system of the lactating infant when from plant origin (Lukasik et al., 2017) or for trans-species effect on adult consumers through dairy products (Baier et al., 2014). The functionality of miRNA is believed to be shared among vertebrates (Brennecke et al., 2005; Xie et al., 2005), more specifically, here, we are assaying functionality of a microRNA common to rat and human. We have chosen rat pup because the model is relevant for studying nutritional programming of the circadian clock (Orozco-Solís et al., 2011). Our general aim was to explore the diffusion in relevant tissues and organs of miRNA delivered by biomimetic vehicles. *In vivo* delivery of miRNA to epithelial cells of the gastrointestinal tract is paving the way to design new supplementation for breastfed babies to put right diet-induced nutritional programming leading to diabetes (Beuzelin and Kaeffer, 2018). The miR-320-3p (also 320a) has been described in exosomes purified from human breast milk (Zhou et al., 2012). A *cis*-regulatory role is known for hsa-miR-320-3p which participates in a negative feedback loop at the polr3d promoter inducing transcriptional gene silencing in Human Embryonic Kidney-293 cells (Kim et al., 2008). Polr3d is the subunit-17 of polymerase-III involved in cell proliferation (Ittmann, 1994) as well as in tumorigenesis (Huang et al., 2017). Due to the natural oral delivery of miRNA-320-3p in the breast milk, we investigated whether miRNA could be delivered orally using a synthetic delivery system. With this goal in mind, we used previously developed lipidic derivatives of natural aminoglycosides shown to be efficient for intracellular delivery of siRNA, DNA, or mRNA (Desigaux et al., 2007; Mével et al., 2012, 2016; Habrant et al., 2016; Colombani et al., 2017). Those lipoaminoglycosides were also shown to be non-toxic in cells (Billiet et al., 2012) and used here for an oral transfer of mature single-stranded miRNA. Single-strand synthetic RNA harboring phosphate group at the 5′ end are known to be bioactive when delivered *in vivo* in target cells (Lima et al., 2012). Moreover, the passenger strand is unnecessary for potent gene silencing (Lima et al., 2012) after cytoplasmic delivery of miRNA. Here, we have tested on rat pups, whether extracellular miRNA complexed with lipoaminoglycosides can be delivered in cytoplasm and nucleus of gastric cells with or without interaction with extracellular vesicles of gastric fluid. It should be underlined that little is known concerning the nuclear delivery of the non-complexed miRNA (Roberts, 2014; Kalantari et al., 2016).

The capacity to deliver miRNA beyond stomach has been assayed in plasma and in cells of different organs (small intestine, liver, inguinal adipose tissue, choroid plexus, and cortex) and according to a daily rhythm. In rodents, the emergence of circadian clock outputs occurs during the first 2 or 3 weeks after birth. The pre and postnatal developments of the molecular clockwork in the rat liver proceed gradually with clock transcript oscillations well-organized after 30 days of life (Sládek et al., 2007). Early rhythm is entrained by the rhythm in breastfeeding and care of the newborns (Sumová et al., 2006). Apparently, before weaning, peripheral clocks’ setting by the feeding regime may prevail upon entrainment by the suprachiasmatic nuclei. Here, we apply miRNA supplementation from Day-12 of age because extensive changes in gene expression of neurodevelopmental process related to cell differentiation and cytoskeleton organization, have been identified in the hypothalamus of rat pups born from low protein-fed mothers (Coupé et al., 2010).

The miR-320-3p has been studied for post-transcriptional gene silencing in the cytoplasm on rat endothelial and cardiac cell cultures derived from diabetes situation [on several genes among which the heat shock protein family B (small) member 6 (Hspb6) or Hsp20 (Montecalvo et al., 2012)]. The hspb6 (also hsp20) gene is highly expressed in several organs including stomach (Liu et al., 2015). In addition, the antagomiR of miR-320-3p has been used to attenuate neurologic injuries after neural chord ischemia (He et al., 2015). Putatively, the *in vivo* delivery of miR-320-3p should target binding sites located both on polr3d promoter as well as on polr3d and hspb6 mRNA in targeted cells.

In this paper, we demonstrate that the lipoaminoglycoside DOSP allows the oral delivery of miR-320-3p with internalization into gastric cells. The exogenous miR-320-3p promotes polr3d transcript expression according to daily rhythm and association of the molecular complex of His3-K4-me3 with the polr3d promoter.

## Materials and Methods

### Lipoaminoglycosides

**FIGURE 1:**
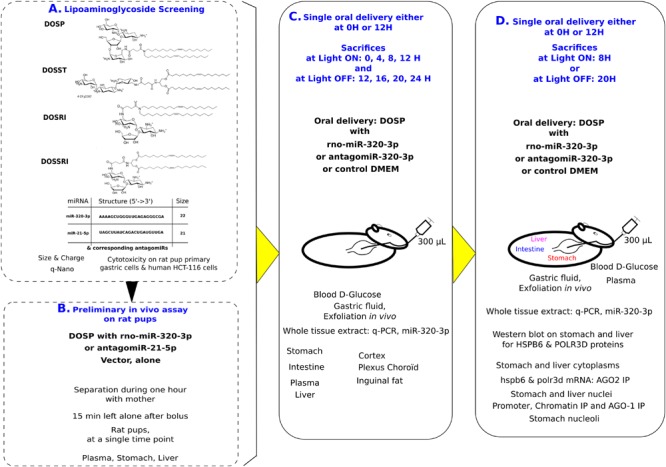
Experimental design: First, we have selected on lack of cytotoxicity and nanoparticle properties, the best vehicle out of four molecules **(A)**. Second, we have selected conditions for *in vivo* oral bolus **(B)**. Third, we realized two experiments with time series over 12 h at light on or off, with miR-320-3p or its antagomiR embedded in dioleyl-succinyl-paromomycin (DOSP) and Control **(C)**. Finally, we have selected the time for inoculation maximizing detection of miR-320-3p to realize *in vivo* experiments according to circadian physiology and comparatively to antagomiR and control groups **(D)**.

The four different lipoaminoglycosides studied herein were dioleyl-succinyl-paromomycin (DOSP), dioleyl-succinyl-serinyl-tobramycin (DOSST), dioleyl-succinyl-ribostamycin (DOSRI), and dioleyl-succinyl-serinyl-ribostamycin (DOSSRI) and were kindly provided by In-Cell-Art (Nantes, France). Formulas are shown in Figure 1A. These vectors with or without ribonucleic acids were prepared extemporaneously in Dulbecco Minimal Essential Medium (DMEM) without antibiotics before incubation with cultured cells. For *in vivo* experiments, the non-cytotoxic vector was prepared extemporaneously in DMEM without antibiotics and given as an oral bolus of 300 μL to breast-restricted rats.

### Tunable Resistive Pulse Sensing (TRPS)

Particle size and charge of each lipoaminoglycoside were assayed by tunable resistive pulse sensing using q-Nano F4-2 (Izon, Billinge et al., 2014). Nanopores 150 and 200 nm were used with corresponding calibration particles. All samples were diluted in Phosphate-buffered solution without Ca^++^ and Mg^++^ containing 0.03% Tween-20. The four lipoaminoglycoside solutions were prepared by mixing 2 μl of DOSP, DOSST, DOSRI, or DOSSRI (kind gift of In-cell-art inc., France) with 98 μl of PBS-Tween-20 (30%). The miRNA concentration range was prepared by mixing miR-control (5, 0.5, or 0.05 nmol diluted in 50 μl PBS-Tween) with DOSP (2 μl dilute in 50 μl PBS-Tween). Data analyses were done on Izon suite 3 software.

### miRNA and AntagomiR

We purchased single-strand synthetic miRNA and corresponding antagomiR, HPLC grade from Eurofins Genomics. The hsa or rno-miR-320-3p: 5′-AAAAGCUGGGUUGAGAGGGCGA-3′, MW 7223, Tm 62.1°C, 22 nucleotides, with its antagomiR-320-3p: 5′-UCGCCCUCUCAACCCAGCUUUU-3′. The rno-miR- 21-5p 5′-UAGCUUAUCAGACUGAUGUUGA-3′, with its anta- gomiR-21-5p 5′-UCAACAUCAGUCUGAUAAGCUA-3′. Each synthetic molecules was checked by q-PCR with TaqMan probes and for antagomiR-320-3p or 21-5p we have checked the effect by *in vitro* assay with gastric or HCT-116 cells of down-regulation of miR-320-3p or miR-21-5p expression, respectively.

### *In silico* Prediction of miRNA Binding Sites

The putative mRNA targets for miR-320-3p were searched by miRWalk and checked by alignments. For RNA polymerase III subunit D (Polr3d, rat – ENSRNOG00000010028; human – ENSG00000168495 also known as RPC4, BN51T, and TSBN51), we have found reverse alignment of miR-320-3p in polr3d promoter both for rat and human and one target site in three prime Untranslated Terminal Region (3′-UTR) corresponding to eight bases of the germinal seed for rat but only six bases of the germinal seed for human. For heat shock protein family B (small) member six (Hspb6, rat – ENSRNOG00000020922; human – ENSG00000004776; also known as FLJ32389, Hsp20, PPP1R91, protein phosphatase 1, regulatory subunit 91), we have found one target site in 3′-UTR corresponding to seven bases of the germinal seed for rat as well as for human. For clock (ENSRNOG00000002175), we have found a target site in 3′-UTR for rno-miR-320-3p. The miR-320-5p sequence is also identical in human and rat. Nomenclature of miR-320 family is summarized elsewhere (Beuzelin and Kaeffer, 2018).

### HCT-116 Cell Line

The HCT-116 cell line was a mycoplasma-free human colon cancer cell line from the laboratory collection.

### Wistar Rats

Rat mothers with twelve rat pups were purchased from Janvier (Le Genest Saint Isle, France) for explantation in gastric primary cultures (*n* = 12 pups), for preliminary *in vivo* experiment (*n* = 12 pups) and for *in vivo* experiments with time series (*n* = 48 pups). Rats with pups were allocated in a room either with a light on at 7:00 am or off at 7:00 pm and allowed to adapt for 5 days before the experiment. Our experimental protocol was approved by the “Comité d’éthique pour l’expérimentation animale, Pays de la Loire, France” under number 06 (January 27, 2017 #APAFIS-8406). Studies on rats were performed according to the rules of the Nantes animal experimental unit [in compliance with the European Communities Directive of 24 November 1986 (86/609/EEC) and the Principles of laboratory animal care (NIH publication no. 85–23, revised 1985)].

### Experimental Design

Experimental design shown on Figure 1 summarizes the four experimental steps. First, we have selected on cytotoxicity assay and nanoparticle properties, DOSP as non-cytotoxic vector out of four molecules (Figure 1A). Second, we have selected our conditions of *in vivo* inoculation (Figure 1B). The antagomiRNA-21-5p or miR-320-3p (0.5 nM corresponding to 9 × 10^10^ molecules given per rat, allowing a pressure of 100 molecules/cell on 9 × 10^8^ gastric cells) was used to explore *in vivo* conditions of inoculation. Third, we realized two experiments with time series over 12 h with miR-320-3p and its antagomiR selecting the best 2 h to explore circadian physiology following oral bolus (Figure 1C). Experiments using Fluorescence energy transfer have shown on immortalized cells that transfected si-RNA are reorganizing crucially between cytoplasm and nucleus during the first 4 h (Hirsch and Helm, 2015), as a consequence, we have chosen this time point as the first time of evaluation during our *in vivo* experiments. Moreover, for proper delivery of ex-miR-223-3p in neighboring cells, many studies have shown that miRNAs are highly stable in most cell types, with a half-life ranging from 28 to 220 h (Zangari et al., 2017); our conditions are using 12 h as the maximum time taking this into account. The fourth and final step, the optimal post-inoculation time was at 8 h allowing maximum detection of miR-320-3p in most organs (Figure 1D). Rat pups were sorted at random and sex was noted. To minimize stress, visual contact was maintained with the mother during the breast-restricted period. Pups were gently put in a separate box with litter and tissue paper to minimize thermic stress for 1 h. Except one pup used as 0 or 12 H untreated control, each received a bolus with a flexible catheter fitted on a 1-mL syringe of either DMEM, miRNA-320-3p or antagomiR-320-3p at 5 nM corresponding to 9 × 10^11^ molecules given per rat, allowing to inoculate 100 molecules in 9 × 10^9^ gastric cells. After marking with indelible ink, they were returned 15 min in a separate box before re-uniting with their mother. Total separation time was of 75 min.

### Cytotoxicity Screening and Detection of Intracellular Accumulation of miR-320-3p in Primary Cultures of Rat Gastric Cells and Human HCT-116 Cells

Cytotoxicity of lipoaminoglycosides was explored by daily microscopic examination on gastric tissue explants and primary gastric cell cultures of rat pups. Exfoliated cells were evaluated after recovery from cell supernatants by centrifugation. Twelve rat pups were used to explant gastric gland *in vitro* as described (Kaeffer et al., 2011). Delivery of miRNA-320-3p in gastric glands was detected by q-PCR, at 8 and 12 H post-transfection. For primary cultures, a stomach of a 12-Day pup was cleaned from milk and gastric fluids, minced into small pieces and digested in 5 volume of trypsin/EDTA (Gibco) for 30 min in 37°C. After 2 min sedimentation, the supernatant was yielded and diluted in 10 ml DMEM-GlutaMax-D-Glucose. Digested tissues were filtered through 200 μm strainer and centrifuged at 1000 rpm for 5 min. The pellet containing cells were seeded in collagen I-coated P-24 well and cultured in 20% fetal calf serum in DMEM-GlutaMax-D-Glucose supplemented medium with 1% penicillin/streptomycin (Gibco). The transfections with DOSP or DOSST were done 1 week later. 30 min before transfection, the medium was removed and replaced by 500 μL of fresh medium without serum.

Transfection solutions were prepared by mixing miR-320-3p (19 pmol diluted in a 50 μl medium) to DOSP or DOSST (2 μl in 50 μl medium according to In-cell-art requirements). The solution was kept at room temperature for 15 min to allow the complexes to form. The entire volume of complexes is added to each well and incubated 3 h before the medium is supplemented with 400 μl of medium supplemented with 50% fetal calf serum. One or 2 days after the miRNA transfection, cells were washed with PBS, lysed in Qiazol and stored at −80°C.

Cytotoxicity of artificial vehicles was also explored on HCT-116 cells seeded at a density of 20,000 cells/wells in black 96-well plates (Nunc) and cultured in 10% fetal calf serum in DMEM-GlutaMax-D-Glucose supplemented medium with 1% penicillin/streptomycin (Gibco) for 2 days at 37°C with 5% CO_2_. 30 min before transfection, the medium was removed and replaced by 100 μL of fresh medium without serum. The four lipoaminoglycoside solutions were prepared by up-down mixing 0.4 μl of DOSP, DOSST, DOSRI, or DOSSRI (In-cell-art, France) with 20 μl of medium without serum and kept for 15 min at room temperature. Then, the transfection solutions were added to cell monolayer for 3 h. After transfection, 100 μl of a complete medium is added to cells. Cell viability was quantified 5, 24, or 48 h post-transfection. The medium was replaced with new media supplemented with 0.1% (v/v) of stock resazurin solution (Sigma) and then incubated for 3 h. Fluorescence intensity was measured using a Varioskan Lux (Thermo Fisher Scientific) at a 560-nm excitation wavelength and a 590-nm emission wavelength.

### Gastric Fluid Fractions and Recovery of Exfoliated Cells From Gastric Fluid

Gastric fluids were centrifuged to pellet exfoliated cells (3,000 × *g* for 10 min, room temperature). Both cell-free supernatants, containing the exosomes for q-Nano analyses, and exfoliated cell pellets were stored −80°C. Biophysical properties and interactions between artificial and natural extracellular vesicles in gastric fluids were studied on q-Nano (Izon) deriving the charge and size of gastric extracellular vesicles.

### Plasma Parameters

The miR-320-3p has been associated with the regulation of Glucose-Induced Gene Expression in Diabetes (Feng and Chakrabarti, 2012). At sacrifice, rat pup was sampled of one blood drop to determine the total blood glucose by AccuChekH Active (Roche-Diagnostics GmbH, Mannheim, Germany), and blood plasma was collected on EDTA tubes (Wang et al., 2012), after centrifugation at 2,000 × *g* for 10 min and stored at −80°C. Cholesterol (Cholesterol FS^*^, DiaSys Diagnostic Systems GmbH), Triglycerides (Triglycerides FS^*^, DiaSys Diagnostic Systems GmbH), and Non-Essential Free fatty acids (NEFA FS^*^, DiaSys Diagnostic Systems GmbH, Holzheim, Germany) were measured on rat plasma.

### Organ Samples

At sacrifice, stomach, small intestine (1 cm below pyloric end), liver, inguinal adipose tissue, and cortex were directly immersed in liquid nitrogen. Plexus choroids were dissected out (Bowyer et al., 2012) under binoculars and immersed in liquid nitrogen.

### Quantitative-Polymerase Chain Reaction (q-PCR)

Mirna assay was done using 3 miRNAs as reference genes (rno-miR-146b-5p, let-7d-5p, let-7g-5p; after adaptation from human (Floris et al., 2015) to rat species) on rat breast milk, gastric fluids (crude, exfoliated cells, and protein fraction), stomach, intestine, plasma, liver, plexus choroid, cortex.

For gene expression analysis, all organs were homogenized in Qiazol buffer (Qiagen) using Precellys tissue homogenizer (Bertin technologies). Total RNA extraction was done like miRNA extraction. After treatment with DNase I (Promega) and reverse transcription of total RNA with high capacity cDNA reverse transcription kit (Applied Biosystems), real-time quantitative PCR was performed using Biorad CFX connect real-time system.

Likewise, mRNA assay was done using 3 reference genes (rno- beta-2-microglobulin, beta-actin, and Usb1). We have taken into account critics of normalization against reference genes (Stevanato et al., 2016) by checking the stability of our reference genes.

The circadian clock was explored with period1, bmal1, clock, npas2, and c-myc. Non-specific binding of miR-320-3p or antagomiR-320-3p was evaluated with stat1. Table 1 gives a complete list of probes purchased from TaqMan or self-designed.

**TABLE 1:** List of probes purchased from TaqMan or self-designed for SYBGreen.

**Transcript**	**Name**	**Supplier and fluorochrome**	**Assay ID**	**Primer (5′–3′)**
miRNA	rno-mir-146b	TaqMan, FAM	Classic-002755	
			Advanced–rno480941_mir	
	let-7d		Classic-002283	
			Advanced–rno478439_mir	
	let-7g		Classic-002282	
			Advanced–rno478580_mir	
	rno-mir-320-3p		Classic-002277	
			Advanced–rno481048_mir	
	rno-mir-320-5p		Advanced–rno481049_mir	
	rno-mir-21-5p		Classic-000397	
	rno-miR-484		Advanced–rno481181_mir	
	rno-miR-20a-5p		Advanced–rno478586_mir	
	primir-320		rn03465856_pri	
	primir-21		rn03464993_pri	
	mRNA		Assay ID	
	usb1		rn01536722_m1	
	b2-microglobulin		rn00560865_m1	
	b-actin		rn00667869_m1	
	ccnd1		rn00432359_m1	
	sirt6		rn01408249_m1	
	sirt1		rn01428096_m1	
	per1		rn01325256_m1	
	polr3d		rn01468090_g1	
	hspb6		rn00594138_m1	
	arntl		rn00577590_m1	
	c-myc		rn00561507_m1	
	npas2	Primer (5′–3′) – self-design for SYBRGreen		FW: CGTCCAGATGTTTCTACAGCAGC
				R: TGGTTTGTGACTTGGGTGGA
	stat1			FW: GAACGTGCTCTGCTCAAGGA
				R: CATGGAAGTCGGGTTCACCT
	Clock			FW: GAACTTGGCTTGAGGAGTCT
				R: GTGATCGAACCTTTCCAGTGC

### Western Blot on POLR3D and HSPB6

Fragments of stomach or liver were homogenized in RIPA buffer (Sigma) containing a cocktail of protease and phosphatase inhibitors (Sigma). Equal amounts of solubilized proteins (20 μg) were loaded on 4–15% gradient Mini-protean TGX gels (Bio-Rad), blotted onto nitrocellulose membranes and incubated with the following primary antibodies: POLR3D (NBP2-56240; diluted at 1/500) from Novus Biologicals, HSPB6 (ab13491; 1/2,000) from Abnova or beta-actin (A5441; 1/2,000) from Sigma. Anti-rabbit or anti-mouse IgG labeled with Dylight 800 or Dylight 680 were used as secondary antibodies. Proteins were determined by Infrared fluorescent detection (LI-COR Odyssey) and quantification was performed using Image Studio Lab v5.2 (LI-COR).

Two molecular forms of POLR3D are described and we have used a rabbit monoclonal antibody (NBP2-56240) allowing detection of both molecular forms in rat or human tissues. Three molecular forms of HSPB6 are described in humans. We found two molecular forms in Rat with rabbit polyclonal antibody (abcam, ab13491) raised against rat skeletal muscle. Anti-beta-actin antibody was used as control. Western blots were realized on stomach and liver protein extracts.

### Promoters

We designed primers to detect promoters of polr3d and marf1 in immunoprecipitation with anti-Ago1 or Chromatin Immunoprecipitation. In human (Kim et al., 2008) and in rat, the miR-320-3p is found in reverse alignment with the polr3d promoter and the miR-484 in reverse alignment in the marf1 promoter. We designed the following rat promoters. Rno-POLR3D-prom:Fw: 5′-CAGACCAGTCACCTCATCCTTT-3′ and Rv: 5′-AGTATTTATCAGACGGTGCCTC-3′; Rno-marf1-prom du rat: Fw: 5′-GATAACCCCCTATTTTGAGGTT-3′and Rv: 5′-GCGTCTTCTCCGCGCAGGGCAT-3′. To evaluate the number of DNA molecules harboring miR-320-3p inverted sequences, we used corresponding primers for miR-320-3p with universal primer (Mei et al., 2012). The iQ SYBR Green Supermix (Bio-Rad) was used to perform real-time PCR on an iCycler iQ system (Bio-Rad) with the promoter or miR-specific primers.

### Cytoplasm, Nucleus, and Nucleole Preparations

Stomach samples were thawed from −80°C, rinsed with 1 ml ice-cold PBS0 and centrifuged 5 min at 4°C and 2,000 × *g*. Samples were resuspended in 500 μL hypotonic buffer [10 mM Tris–HCl, pH 7.4; 10 mM KCl, 1.5 mM MgCl_2_, 10 mM NaCl, 0.15 mM EGTA, anti-Protease/phosphatase (with EDTA) + 0.5% NP-40]. Tissues were grinded with Potter at 5,000 rpm for 1 min on ice. We added 25 μL NP-40, 10% and vortexed at maximal speed for 10 s. Samples were left 10 min on ice, and centrifuge 5 min at 300 × *g*, 4°C. The supernatant is the cytoplasm fraction and the pellet corresponds to a nucleic fraction.

The cytoplasmic fraction is centrifuged 10 min at 800 × *g* and subdivided in an RNA and protein tubes. RNA tube is precipitated by Qiazol for a minimum of 1 h at −20°C. One ml RIPA is added to the Protein tube before storage at −20°C.

For nucleic fraction the pellet is resuspended with 500 μL hypotonic buffer for nuclear fraction [10 mM Tris–HCl, pH 7.4; 10 mM KCl, 250 mM sucrose, 1 mM DTT, 3 mM MgCl_2_, 10 mM NaCl, 0.15 mM EGTA, anti-Protease/phosphatase (with EDTA) + 0.5% NP-40]. The suspension is vortexed 10 s at maximal speed, then centrifuged 5 min, 4°C at 500 × *g*. The pellet is resuspended in 300 μL hypotonic buffer for nuclear fraction and carefully pipetted on top of a cushion of 300 μL of sucrose buffer (0.35 M; S2 buffer) and centrifugation for 5 min at 1430 × *g* and 4°C. The pellet is resuspended in 300 μL S2 buffer. Presence of nuclei was checked by fluorescent microscopy with Dapi (4′,6-diamidino-2-phénylindole) labeling. The nuclei fraction is subdivided in an RNA tube, precipitated by Qiazol for a minimum of 1 h at −20°C or a Protein tube, in which one ml RIPA was added before storage at −20°C.

For extracting nucleoli, the nuclei were sonicated on ice (amplitude 50%, 4 × 10 s with 15 s lag between each sonication cycle). Destruction of nuclei was checked under a fluorescent microscope with DNA labeling by Dapi. In a new 1.5 ml tube, we added 300 μL sucrose buffer (0.88 M, buffer S3) and gently put the sonicated sample on top of buffer S3 and centrifuge 10 min at 2800 × *g*, 4°C. The pellet and the supernatant contained nucleoli and nucleoplasmic fraction, respectively. The pellet was rinsed once with sucrose buffer S2 (0.35 M) and centrifuged again 5 min at 2800 × *g*, 4°C. Nucleoli pellet was stored at −80°C.

### Immunoprecipitation by Anti-AGO1 or 2 on Cytoplasm or Nucleus Lysates

The procedure was adapted after (Turchinovich and Burwinkel, 2012). For immunoprecipitation with anti-AGO2, stomach or liver tissue samples were thawed from −80°C, rinsed with 1 ml Phosphate-buffered solution without Ca^++^ nor Mg^++^ and lysed by 600 μL Pierce^TM^ IP lysis buffer (Thermo Fisher Scientific #87788) on ice for 5 min with periodic vortexing. The cellular lysate was centrifuged 10 min, 4°C at 13,000 × *g*, and 150 μL supernatant was incubated overnight at 4°C with anti-AGO2 Rabbit Monoclonal Antibody (R.386.2; Thermo Fisher Scientific).

Then, samples were incubated with 20 μL Pierce^TM^ Protein A/G Plus Agarose (Thermo Fisher Scientific # 20423) at room temperature for 2 h with shaking. Samples were rinsed twice with 1 ml Pierce^TM^ IP lysis buffer. Complexes captured by the resin were resuspended in Qiazol and processed for RNA/DNA extraction. For immunoprecipitation with anti-AGO1, we used a similar procedure, lysing nucleus prepared from stomach or liver and incubating with anti-AGO1 Antibody (6H1L4, Thermo Fisher Scientific).

### Chromatin Immunoprecipitation With Anti-trimethylated Histone-3 K4 or K27

Chromatin immunoprecipitation was performed with Pierce Chromatin Prep Module (Thermo Scientific #26158). Briefly, small tissue aliquotes were cross-linked by exposition to 1% formaldehyde. Chromatin was fragmented by Micrococcus Nuclease.

Immunoprecipitations were performed using 1 μg of Anti-Trimethyl-Histone-3-Lys-4 (-Thermo Fisher Scientific; catalog # PA5-17420) or Anti-Trimethyl-Histone-3-Lys-27 (-Thermo Fisher Scientific; catalog # MA5-11198) overnight at 4°C. Micrococcus nuclease was used at 0.25 μL/sample.

We collected immune complexes with agarose A/G for 2 h at 4°C, beads were rinsed twice by PBS0 and pelleted at 94 × *g* for 1 min. Immune complexes were eluted by adding 100 μL of elution buffer to pelleted beads. After brief vortexing, preparations were incubated at Room Temperature for 15 min. Thereafter, beads were spun down and the supernatants (eluates) carefully transferred to another tube. The elution step was repeated. Both eluates were combined.

We added 5 M NaCl and proteinase K allowing crosslink reversion by 1.5-h incubation at 65°C.

Nucleic acids were recovered by Qiagene miRNA-Easy kit and analyze ChIPped chromatin using quantitative PCR. iQ SYBR Green Supermix (Bio-Rad) was used to perform real-time PCR on an iCycler iQ system (Bio-Rad) with promoter-specific primers.

### Statistical Analysis

We used two-way ANOVA with interaction for all *in vivo* analyses. We defined the Day/Night factor as the first factor, corresponding to the two experimental settings: 8 h after light on or off. The second factor, defined as Treatment factor, was the three oral bolus combinations: miR-320-3p, antagomiR-320-3p (simplified as antagomiR), and control groups. We have shown the probability value corresponding to the null hypothesis tested according to factor-1, factor-2 or, interaction. *Post hoc* comparisons were also performed when needed between groups. We have checked the equivalence between delta-delta-Cq expression (Livak and Schmittgen, 2001) and fold change that is used in this paper. Fold changes were calculated either according to the control rat pup at 0 H for 4, 8 and 12 H series or 12 H for 16, 20, and 24 H series. For comparing groups, we defined fold changes as the ratio between the single value of q-PCR or of Western blot on the average of Control group at ZT-8H. Statistical analyses were performed using GraphPad Prism (GraphPad Software v.6.0) or R (Fox, 2005). Fold change data were chosen over delta-delta-Cq and Log transformed when appropriate for simplicity of understanding and graphical purposes. The normality of distribution tested by Shapiro–Wilks of Delta-delta-Cq data was improved by the Log-transformation, justifying our use of ANOVA for the comparison between groups.

## Results

### Biophysical Analysis of Lipoaminoglycoside by Tunable Resistive Pulse Sensing (TRPS)

**FIGURE 2:**
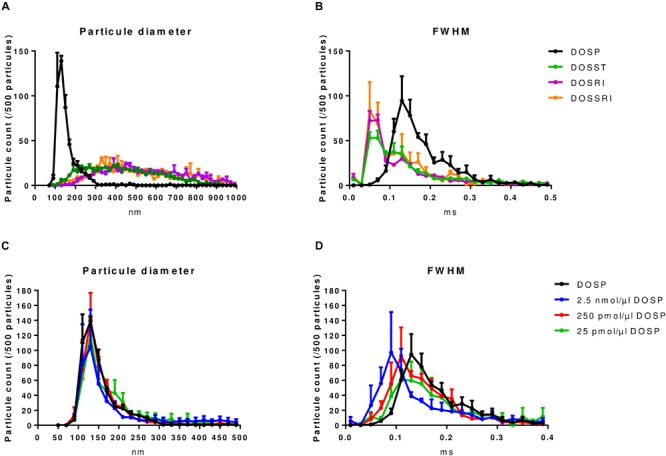
Physicochemical characterization of lipoaminoglycoside alone and DOSP/miRNA by tunable resistive pulse sensing. **(A)** Particle size distribution of dioleyl-succinyl-paromomycin (DOSP), dioleyl-succinyl-serinyl-tobramycin (DOSST), dioleyl-succinyl-ribostamycin (DOSRI), and dioleyl-succinyl-serinyl-ribostamycin (DOSSRI). **(B)** Full width half maximum (FWHM) of the four molecules. Note the sharp peak for DOSP at 150 nm with the slower transit time suggesting a more positive particle surface charge. We did not find any effect of ribonucleic acid loading on DOSP size **(C)**, but a slowing effect according to ribonucleic acid concentrations on FWHM **(D)**. DOSP/miRNA complexes with charge ratios ± of 0.043, 0.43, and 4.3. Each point and error bar represent the mean of four experiments.

Prior to *in vitro* and *in vivo* studies, we assessed the physicochemical properties of complexes resulting from the association of the lipoaminoglycosides with a given miRNA compared to those obtained without miRNA (Figure 1A). Tunable resistive pulse sensing method was used to measure the size of complexes. In the absence of miRNA, DOSST, DOSSRI, and DOSRI particles did not display any specific particle diameter (heterogeneous diameter ranging from 90–1000 nm), except for DOSP particles displaying a peak at about 150 nm (Figure 2A). The full-width half maximum (FWHM) value is related to the nanoparticle surface charge. The smaller the FWHM is, the more it is negatively charged. DOSP had a more positive particle surface charge (FWHM peak at 0.17 ms) compared to DOSST, DOSRI, and DOSSRI (FWHM peak at 0.05 ms; Figure 2B).

Dioleyl-Succinyl Paromomycin nanoparticles, unloaded or loaded with different concentrations of ribonucleic acid (25 pmol to 2.5 nmol/μl DOST; Figure 2C) which corresponds to charge ratios ± ranging from 4.3 to 0.043. DOSP nanoparticles had a mean diameter of 125 nm irrespective of the miRNA amounts (Figure 2C). FWHM analysis of lipoaminoglycosides alone shows that particles of DOSP had a pike of 0.13 ms corresponding to a 2 fold increase compared to that of DOSST, DOSSRI, and DOSRI. The FWHM of DOSP/miRNA complexes increased from 0.09 to 0.13 ms when the DOSP/miRNA charge ratio ± was raised from 0.43 to 4.3 (Figure 2D). This strongly suggests that miRNA molecules were fully entrapped with a DOSP/miRNA charge ratio ± of 4.3 as the same FWHM was obtained with DOSP alone. Of note, particle size observed with DOSP is similar to that of natural extracellular vesicles expressing CD63 (100–200 nm), with a peak at 130 nm (Data not shown).

### *In vitro* Screening of Lipoaminoglycosides

Ionizable lipids improve penetration of nucleic acids through cell membrane but can also display some cytotoxicity. Thus before conducting any gastric inoculation, cytotoxicity of the four lipoaminoglycosides was examined. On HCTT116 cells, only DOSRI nanoparticles decreased cell viability 48 h post-transfection (Supplementary Figure S1A), but DOSRI and DOSSRI were found cytotoxic on gastric gland explants and primary cultures of rat gastric epithelial cells following microscopic examination with strong rapid acidification of the culture medium (data not shown). Consequently, only DOSP and DOSST were considered non-cytotoxic.

Next, we investigated if DOSP and DOSST were efficient for transferring miRNA into gastric cells. Gastric primary cultures cells were transfected with DOSST or DOSP loaded or not with 9.5 pmol of miR-320-3p. The miR-320-3p levels in cells transfected with DOSP/miR complexes were much higher than those transfected with DOSST/miR complexes. The miRNA levels were stable from 24 to 48 h (Supplementary Figure S1B). The transfection of DOSP and DOSST alone did not change the intracellular level of mir-320-3p. The same experiment was carried out in gastric glands and gastric exfoliated cells only with DOSP. Cells were harvested 8 and 12-h post-transfection. A strong increased of miR-320-3p levels occurred in gastric glands and exfoliated cells transfected with DOSP/miR complexes, whereas no modification was observed with DOSP alone (Supplementary Figure S1C). These results show that DOSP nanoparticles allowed for rapid and efficient miRNA delivery in cultured cells, so it was selected for the *in vivo* studies.

### Tracking miR-320-3p in Gastric Fluids, Gastric Exfoliated Cells, and Organs

We did a preliminary *in vivo* experiment of an oral bolus of microRNA complexed DOSP in order to assay the effect of miRNAs and empty DOSP on rat pup physiology (Figure 1B). We used 10 pmol/μl DOSP with miR-320-3p or antagomiR-21-5p for oral treatment and pups were sacrificed 6 h post-bolus. On stomach, we have found only a 1.6 fold increase of mir-320-3p level and a decrease of 50% with antagomiR-21-5p (unshown data). We did not observe any effect on the liver. The preliminary *in vivo* experiment showed that higher miRNA concentration was required to expect an effect on RNAi and promoter association.

Thereafter, we performed two experiments with time series over 12 h (one at light-on, one at light-off), by giving an oral bolus of miR-320-3p complexed with DOSP at a ratio of 250 pmol miRNA/μL DOSP, antagomiR corresponding complex, or control medium. The first experiment allowed to explore 0, 4, 8, 12, 16, 20, and 24 H with ZeitGeber (ZT) at the light on using one or two pups per time (Supplementary Figure S2). Time series evaluation over 12 h was realized on the stomach wall and on six additional organs (small intestine, blood plasma, liver, inguinal adipose tissue, plexus choroid, and cortex) as shown in Supplementary Figure S2. Under our hands, rno-miR-320-5p in Wistar rat breast milk, stomach or cortex was below detection level by q-PCR. To our knowledge, the miR-320-5p has been detected only in mouse pancreas using RNA sequencing (Mo et al., 2017). The highest detection of miR-320-3p was obtained on the stomach, with the group receiving miR-320 complexed DOSP, at 8 h after inoculation (ZT-8H and ZT-20H). In the small intestine, the highest detection was obtained at 4 h after inoculation (ZT-4H and ZT-16H) and we obtained a high heterogeneity of detection with plexus choroid. With the group receiving antagomiR-DOSP, we have obtained very low levels of detection for miR-320-3p at ZT-4H or ZT-16H on the stomach, exfoliated cells, and small intestine. On plasma, the low level of detection was obtained only at ZT-4H. Future experiments searching for an optimized effect of antagomiR would be better realized at 4 h after inoculation (ZT-4H and ZT-16H).

**FIGURE 3:**
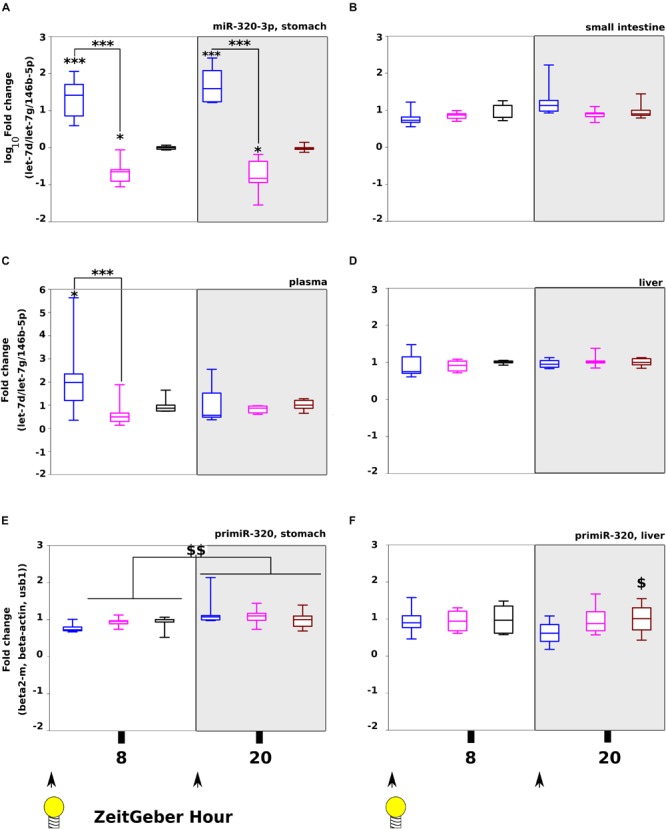
Tracking miR-320-3p in rat pup organs after loading at light on (ZT-8 H) or off (ZT-20 H). Levels of miR-320-3p in the stomach **(A)**, small intestine **(B)**, plasma **(C)**, or liver **(D)** according to a bolus with mir-320-3p (blue box border), antagomiR-320-3p (magenta) and control (black). In parallel, the levels of primir-320 in the stomach **(E)** and the liver **(F)** were determined. We have found highly significant loading of miR-320-3p for both miRNA-supplemented groups in the stomach, 8 h after bolus. A significant loading with miR-320-3p was detected only in plasma at ZT-8H. Note that primiR-320 levels in the stomach was different between ZT-8H and ZT-20H for all groups but not in the liver. In the box plots, a black line within the box marks the median. The boundary of the box closest to zero indicates the 25th percentile and the boundary of the box farthest from zero indicates the 75th percentile. Whiskers above and below the box indicate the 10th and 90th percentiles. We had 5–6 rat pups per group. ^*^P < 0.05 and ^∗∗∗^*P* < 0.001 compared to Controls. ^$^*P* < 0.05 antagomiR-320-3p against control. ^$$^*P* < 0.01 ZT-8H compared to ZT-20H. At the bottom, black arrow-heads remind time of bolus (either at ZT-0H or ZT-12H) and Light bulb that light-on is taken as synchronizer (ZeitGeber), ZT-0H.

Our aim was to maximize the effect of miR-320-3p, consequently, the second experiment was done on 5–6 rat pups per time at ZT-8H and ZT-20H (Figure 1D). The groups receiving the miR-320-3p *per os* at 8 and 20 H had a significantly higher amount of miR-320-3p in the stomach than control or antagomiR (Figure 3A, *p* < 0.001; by two-way-ANOVA Supplementary Table S1). By comparison, we did not find any evolution of miR-320-3p in the small intestine (Figure 3B) or liver (Figure 3D) but we observed a significant difference between miR-320-3p and antagomiR in plasma (Figure 3C, *p* < 0.01) at ZT-8H confirming our previous results. An interaction between Day/Night Factor and Treatment Factor was found at *p* = 0.02, suggesting that blood transfer of miR-320-3p or its antagomiR was dependent on gastric mucosa chronobiology.

In gastric fluids of the three groups, we explored by TRPS, the size distribution of extracellular vesicles in protein fraction over 12 h, every 4 h (Supplementary Figure S3). We did not find striking differences between groups. Likewise, the miR-320-3p level in protein fractions of gastric fluids measured by q-PCR was not different between groups (Supplementary Figure S4). This suggests that mRNA molecules were not associated with extracellular vesicles. As shown in Supplementary Figure S5, we found that the heterogeneity of miR-320-3p was maximum for the group supplemented with miR-320-3p at ZT-8H and ZT-20H both in nucleus and cytoplasm isolated from gastric cells.

We had also evaluated glycemia of pups every 4 h, showing hypoglycemia for the miR-320-3p group (Supplementary Figures S6A,B). Pooling data and samples obtained on *in vivo* experiments at ZT-8H and ZT-20H, we have measured glycemia (Supplementary Figure S6C), cholesterol (Supplementary Figure S6D), triglycerides (Supplementary Figure S6E), and non-essential fatty acids (Supplementary Figure S6F). Results show day/night differences in glycemia (Factor Day/Night, *p* < 0.001) and some hypoglycemia with miR-320-3p complexed DOSP corresponding to an hyperglycemia for the antagomiR group (Factor Treatment at *p* = 0.09, no interaction). By adding Sex as a third factor, we did not find a difference between male (*n* = 19) and female (*n* = 14). However, we had unequal number of pups per treatment combination calling for a future experiment dealing with the difference between sexes by following physiological effects over several day/night cycles. We have found for triglycerides day/night differences (Factor Day/Night, *p* < 0.001), but no effect of Factor Treatment, nor of interaction. The levels of cholesterol and non-essential fatty acids were not affected by the miRNA treatment nor the Day/Night factor.

Taken together, the results demonstrate that DOSP allows the oral delivery of miR-320-3p into cells of the stomach wall, with some transfer toward plasma or liver. Comparatively to Control groups at ZT-8H or ZT-20H, we had an increase of D-Glucose levels with antagomiR groups and a decrease with miR-320-3p groups.

### Effect With Time Series of miR-320-3p or AntagomiR-320-3p on Transcripts Linked to the Circadian Clock or Non-specific Activation

**FIGURE 4:**
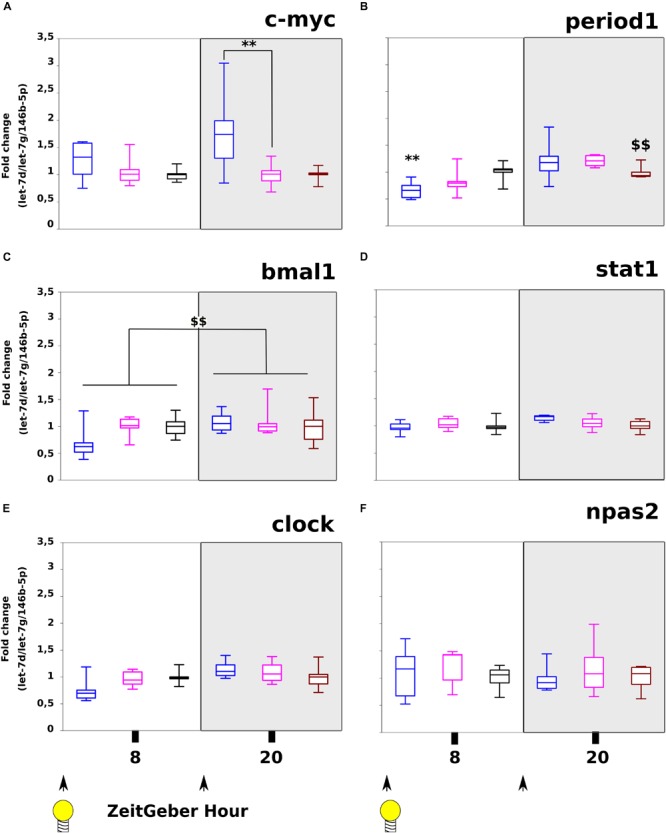
Evaluation of c-myc, circadian clock transcripts and stat-1 following miR-320-3p or antagomiR oral delivery. The mRNA levels of c-myc **(A)**, period1 **(B)**, bmal1 **(C)**, stat1 **(D)**, clock **(E)**, and npas2 **(F)** were assayed in stomach according to a bolus with mir-320-3p (blue box border), antagomiR-320-3p (magenta), or control (black box border). In the box plots, a black line within the box marks the median. The boundary of the box closest to zero indicates the 25th percentile and the boundary of the box farthest from zero indicates the 75th percentile. Whiskers above and below the box indicate the 10th and 90th percentiles. We had 5–6 rat pups per group. ^∗∗^*P* < 0.01 compared to DMEM rats. ^$$^*P* < 0.01, ZT-8H compared to ZT-20H. According to *in vitro* description of polr3d regulation, we found significant evolution of c-myc **(A)** between Day/Night factor (*p* = 0.0342) and highly significant difference between miR-320-3p and antagomiR-320 at ZT-20H (*p* = 0.0144). The period1 transcript **(B)** was significantly different for Day/Night factor (*p* = 0.0001) without any difference between oral bolus combinations but an interaction for miR-320-3p (*p* = 0.0142). The bmal1 **(C)** transcript was only significant for Day/Night factor (*p* = 0.0081). With stat1, we have found a trend for Treatment factor (*p* = 0.062). No evolution was seen with clock **(E)** nor npas2 **(F)**. At the bottom, black arrow-heads remind time of bolus (either at ZT-0H or ZT-12H) and Light bulb that light-on is taken as synchronizer (ZeitGeber), ZT-0H.

On c-myc (Figure 4A), we have found significant differences between ZT-8H and ZT-20H for Day/Night factor (*p* = 0.03) and highly significant difference between miR-320-3P and antagomiR groups at ZT-20H (*p* = 0.01). For evaluation of circadian rhythm in the stomach, we used period1 showing a strong difference between controls at ZT-8H and ZT-20H (*p* < 0.01; Figure 4B) and a highly significant down-regulation for the miR-320-3p group at ZT-8H comparatively to control (*p* < 0.01; Figure 4B). The significant interaction (*p* < 0.05) between Day/Night Factor and Treatment Factor suggests chronobiological dependency for miR-320-3p as well as for antagomiR groups.

Expression of bmal1 was only related to the day-night difference of all groups (*p* < 0.01; Figure 4C). The clock and npas2 transcripts were unchanged on all groups (Figures 4E,F and Supplementary Table S2); in contradiction with our *in silico* prediction for clock. As shown in Supplementary Figure S7, we have found a significant decrease of primiR-21 in the stomach at ZT-20H for the antagomiR-DOSP group (Supplementary Figure S7A). However, we did not find any difference with miR-21-5p (Supplementary Figure S7B), nor in the liver for primiR-21 (Supplementary Figure S7C) or miR-21-5p (Supplementary Figure S7D), neither in the stomach for sirt-1 (Supplementary Figure S7E) and sirt-6 (Supplementary Figure S7F), and cyclin-D1 (Supplementary Figure S7G) between all groups. In the liver, we did not find a difference for c-myc along with stat1, per1 (Supplementary Figure S8), the heterogeneity found with miR-320-3p and antagomiR groups may indicate an effect at distance from inoculation site that should be addressed with further experiments.

For evaluation of the non-specific binding of miR-320-3p or its antagomiR-320-3p, we used the expression of stat1. We found a highly significant effect of Day/Night Factor for stat1 (*p* < 0.01; Figure 4D) with a trend for miR-320-3P supplemented group (Interaction factor at *p* = 0.0662).

### Effect With Time Series of miR-320-3p or AntagomiR-320-3p on Transcript or Protein Levels of polr3d and hspb6

**FIGURE 5:**
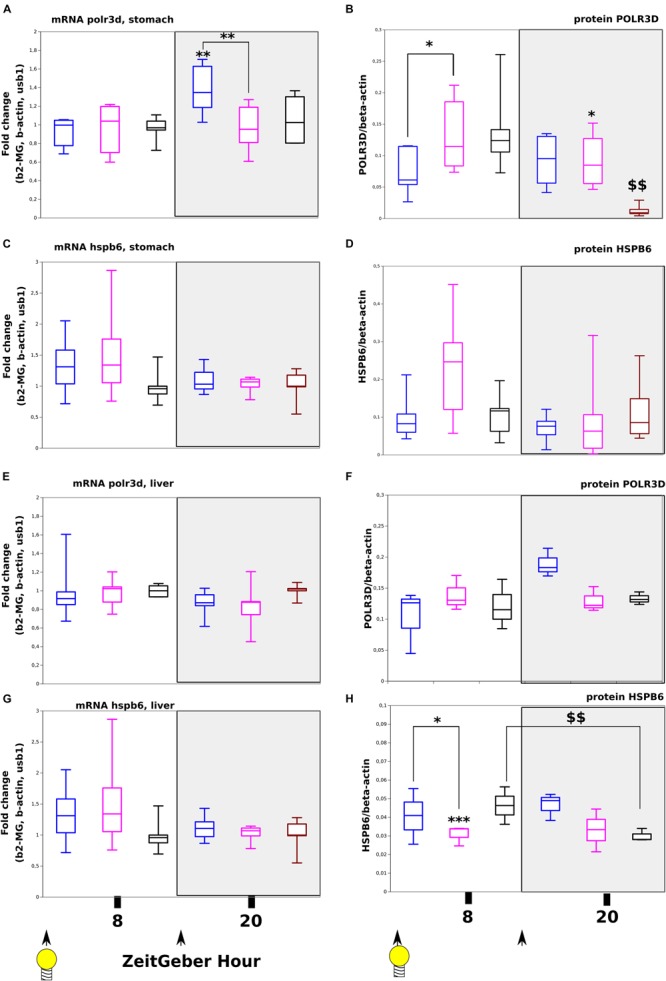
Expression at transcript and protein levels of polr3d and hspb6, target genes of miR-320-3p in rat pups receiving *per os*, miR-320-3p or antagomiR-loaded DOSP nanoparticles or control buffer. Levels of polr3d mRNA **(A)** and protein **(B)** in the stomach according to a bolus with mir-320-3p (blue box border), antagomiR-320-3p (magenta), or control (black box border). Levels of hspb6 mRNA **(C)** and protein **(D)** in the stomach. Levels of polr3d mRNA **(E)** and protein **(F)** in the liver. Levels of hspb6 mRNA **(G)** and protein **(H)** in the liver. B-actin was used as Western blot loading control (*n* = 5–6 per group). In the box plots, a black line within the box marks the median. The boundary of the box closest to zero indicates the 25th percentile and the boundary of the box farthest from zero indicates the 75th percentile. Whiskers above and below the box indicate the 10th and 90th percentiles. We had 5–6 rat pups per group. ^*^P < 0.05 and ^∗∗^*P* < 0.01 compared to DMEM rats. ^∗∗∗^*P* < 0.001 antagomiR-320-3p against control. ^$$^*P* < 0.01 8 ZT-H compared to 20 ZT-H. At the bottom, black arrow-heads remind time of bolus (either at ZT-0H or ZT-12H) and light bulb that light-on is taken as synchronizer (ZeitGeber), ZT-0H.

We have evaluated at the transcriptional and translational levels, the expression of two known miR-320-3p target genes. In the stomach, we found by two-way-ANOVA, a significant increase in polr3d mRNA only at ZT-20H (Figure 5A, *p* < 0.01) but we did not find any evolution for hspb6 both at mRNA and protein levels (Figures 5C,D and Supplementary Table S3). At the protein level on Figure 5B, we have found a highly significant down-expression of POLR3D at ZT-20H (*p* < 0.01) and also a significant down-expression of POLR3D for ZT-8H (*p* < 0.05 comparatively to antagomiR group) and a relative increase of expression for ZT-20H (*p* < 0.05 between antagomiR and control groups). The POLR3D protein level was different between ZT-8H and ZT-20H suggesting daily regulation. On Supplementary Figure S9, we present Western blot bands corresponding to all groups for POLR3D, HSPB6, and beta-ACTIN. We did not observe any difference between groups for the levels of isoform bands for POLR3D as well as HSPB6.

By comparison in liver, we did not find any significant evolution in polr3d both at mRNA and protein levels (Figures 5E,F) as well as for hspb6 mRNA (Figure 5G) except for the expression of HSPB6 protein at ZT-8H showing a strong down-regulation for antagomiR group (Figure 5H; *p* < 0.001). The HSPB6 protein level was statistically different between ZT-8H and ZT-20H suggesting again a circadian regulation in rat pup (*p* < 0.01). These results were compared with cultured HCT-116 cells obtaining no detection of HSPB6 protein with a significant decreased of POLR3D protein with cells exposed to miR-320-3p (unshown results). Preliminary experiments on HCT-116 included miR-21-5p and antagomiR-21-5p (unshown results).

### Evaluation of miR-320-3p in Cytoplasmic AGO2 or Nuclear AGO1 Complexes and in Nucleoli

To be functional miRNAs need to be loaded in the RISC complex build up mainly on Argonaute-2 (in the cytoplasm and related to RNA interference) or on Argonaute-1 (in the nucleus and related to epigenetic regulation of promoter).

Immunoprecipitation with anti-AGO2 showed a lower level of miR-320-3p in immune complexes from pups having received the miR-320-3p bolus (at ZT-8H: delta-Cq miR-320-3p relatively to let-7d-5p and let-7g-5p was of 1.98 against 3.38 for control and at ZT-20H: delta-Cq was of 1.55 against 3.67).

Subsequently to the loading of miR-320-3p in cells, the nucleoli were found to contain an increased amount of miR-320-3p at ZT-20H (at ZT-8H: delta-Cq miR-320-3p relatively to let-20a-5p and let-7g-5p was of 8.68 against 11.97 for control and at ZT-20H: delta-Cq was of 19.29 against 4.94).

These data show that after delivery, miR-320-3p can efficiently integrate the RISC complexes in the cytoplasm with some retro-transfer to the nucleus. In the nucleus, miR-320-3p/Argonaute-1 is slightly enriched at the promoter of polr3d at ZT-20H (Supplementary Figure S10).

### Differences Between Trimethyl Histone-3 K4 or K27 Immune Complexes in Gastric Cells According to miR-320-3p or AntagomiR Supplementation

**FIGURE 6:**
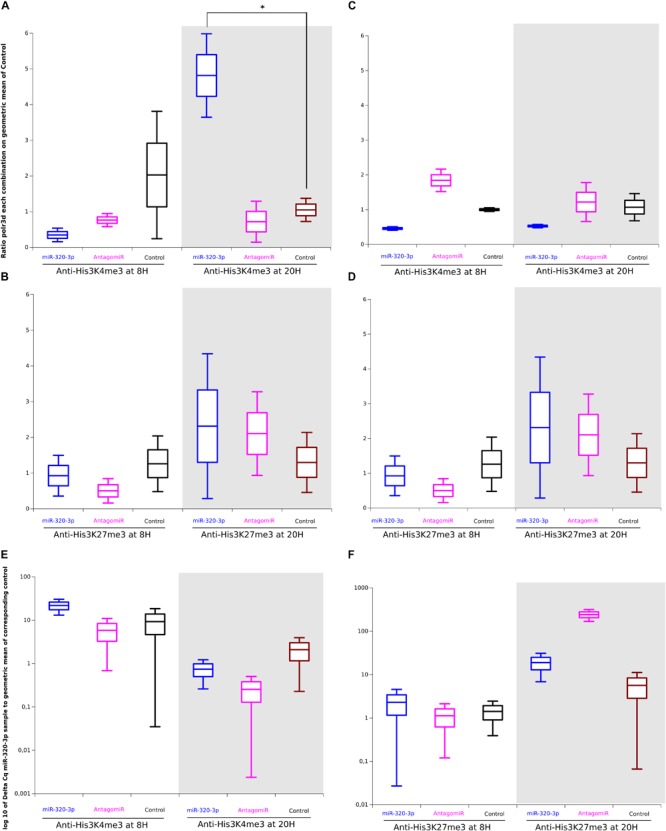
Difference between Chromatin-ImmunoPrecipitation done with anti-Trimethyl-histone-3-Lys4 or Lys-27 on gastric tissues of rat pups treated *per os*, with miR-320-3p or antagomiR-loaded DOSP nanoparticles or control buffer. **(A)** Note a higher ratio of polr3d for miR-320-p group against Control group (^*^P = 0.05) at ZT-20H and the similarity of levels for AntagomiR and Control groups. In panel **(B)** parallel determinations of immune complexes H3K27me3 showed that only the antagomiR-320-3p group was slightly enriched in the polr3d promoter. The immune complexes for H3K4me3 The immune complexes for H3K4me3 at ZT-8H or for H3K27me3 at ZT-20H were enriched in miR-320-3p for rat pups receiving an oral bolus of miR-320-3p as well as for H3K27-me3 for antagomiR-320-3p group at ZT-20H. We used as control the marf1 promoter showing no significant difference in immune complexes for H3K4me3 **(C)** or for H3K27me3 **(D)**. We did not find a significant difference in the relative ratio of miR-320-3p between immune complexes obtained for H3K4me3 **(E)** or for H3K27me3 **(F)**. In the box plots, a black line within the box marks the median. The boundary of the box closest to zero indicates the 25th percentile and the boundary of the box farthest from zero indicates the 75th percentile. Whiskers above and below the box indicate the 10th and 90th percentiles. We had two rat pups per group.

To see whether nuclear miR-320-3p could be associated with the polr3d promoter, we have realized experiments of chromatin immunoprecipitation on stomach preparation incubated with anti-Trimethyl-Histone-3-K4 or Trimethyl-Histone-3-K27. The promoter polr3d was found in higher amount in immune complexes H3K4me3 recovered at ZT-20H from the miR-320-3p group (Figure 6A, *p* = 0.05). Parallel determinations of immune complexes H3K27me3 showed that only the antagomiR-320-3p group was slightly enriched in the polr3d promoter (Figure 6B). The immune complexes for H3K4me3 at ZT-8H or for H3K27me3 at ZT-20H were enriched in miR-320-3p for rat pups receiving an oral bolus of miR-320-3P as well as for H3K27-me3 for the antagomiR-320-3p group at ZT-20H (Figure 6B). We used as control the marf1 promoter showing no significant difference in immune complexes for H3K4me3 (Figure 6C) or for H3K27me3 (Figure 6D). We did not find a significant difference in the relative ratio of miR-320-3p between immune complexes obtained for H3K4me3 (Figure 6E) or for H3K27me3 (Figure 6F and Supplementary Table S4). Using miR-320-3p specific primer and universal primer, we checked for a ratio of 1 between Cq obtained with polr3d against miR-320-3p. Parallel experiments done on the liver did not show any difference between groups.

Our results show that depending on rat pup circadian rhythm, miR-320-3p enrichment in gastric cells is high enough to increase the association between H3K4me3 complexes and polr3d promoter at ZT-20H.

## Discussion

We demonstrate that the lipoaminoglycoside DOSP allows the oral delivery of miR-320-3p with internalization into gastric cells. The exogenous miR-320-3p promotes polr3d transcript expression according to daily rhythm and association between the His3-K4-me3 molecular complex and the polr3d promoter.

The delivery of miRNA orally represents a key challenge as nucleic acids are not stable in a gastric environment. We show here that the lipoaminoglycoside DOSP is non-toxic both on primary cultures and gastric gland explants (Supplementary Figure S1). Physico-chemical characterization of DOSP/miRNA particles showed that the size and the charge were similar to natural exosomes opening the possibility to deliver miRNA in the cytoplasm of cells from gut and blood compartments. As shown on Figure 3 and Supplementary Figure S2, we are using miR-320-3p to track *in vivo* transfection in cells of different organs of a single-bolus of high concentration of synthetic molecules corresponding to mature miRNA, encapsulated in DOPS. Both exfoliated and resident gastric cells were found loaded with a high amount of miR-320-3p (Supplementary Figures S1C, S2B). In addition, we did not find any miR-320-3p accumulation in the protein fraction of gastric fluids (Supplementary Figure S4), nor any effect on vesicular traffic, hours following the oral inoculation (Supplementary Figure S3). We have found different amount of miR-320-3p in plasma only at ZT-8H and between miR-320-3p and antagomiR groups (Figure 3C). The observation implies that the exosome machinery of epithelial, nervous or immune cells from the stomach wall was not induced to secrete higher amount of Extracellular Vesicles in plasma nor in gastric fluids. We are confirming the endosomal escape mechanism described for our vector (Le Bihan et al., 2011). A limitation of our experimental procedure was to optimize the time of delivery according to the detection of miR-320-3p (at 8 h post-inoculation for miR-320-3p-supplemented pups). Consequently, the antagomiR group is clearly not at its optimal timing of delivery (peak at 4 h post-inoculation, Supplementary Figure S2A and Supplementary Table S5).

The miR-320-3p has a strong seeding site in 3′-UTR of hspb-6 mRNA in mouse (Ren et al., 2009), but not in human, and a strong seeding site in the polr3d promoter for rodents and human (Kim et al., 2008). However, conserved sites of binding on mRNA for miR-320-3p *in vivo* may be different from *in vitro.* For instance, alternative cleavage as well as polyadenylation can remove regulatory sites from the message. The phenomenon is apparently widespread in proliferating cells. Proliferating cells harbor shorter UTRs with only half the number of conserved miRNA sites as observed in the longer isoforms that dominate in non-proliferating cells (Vasudevan et al., 2007; Vidigal and Ventura, 2015). In circumstances that induce cells to become quiescent, miRNA targeting of UTRs is reported to enhance rather than repress translation (Vasudevan et al., 2007), more specifically if the UTR is harboring multiple UAUUAUU like on polr3d mRNA with the binding site on miR-320-3p being AAUAAU. Loading gastric cells with miR-320-3p is increasing the mRNA expression of polr3d at ZeitGeber Time 20H (Figure 5A), with a corresponding increase in POLR3D protein both for miR-320-3P and antagomiR (Figure 5B). However, we did not find any difference for transcripts at ZT-8H (Figure 5A) and a significantly lower amount of POLR3D protein (Figure 5B). These results can be explained because we have sampled total stomach wall, containing multiple cell lineages (from epithelium, vasculature, smooth muscle, and immune system); all in a different state of proliferation. MicroRNAs are believed to oscillate between repression and activation in coordination with the cell cycle. In proliferating cells, MicroRNAs repress translation, whereas in G 1/G 0 arrest (which often precedes differentiation), they mediate activation (Vasudevan et al., 2007). Consequently, we have measured the resulting signal for cyclin-D1 transcripts in the stomach. However, we did not find any difference between groups for cyclin-D1 transcripts. Next, we have designed experiments to detect multiple isoforms of proteins with Western blot both for POLR3D and HSPB6. In the stomach and liver, we did not find differences between isoforms for all groups.

Surprisingly, the circadian machinery was deeply impacted. On Figure 4, the mRNA levels of c-myc (Figure 4A) was altered according to extracellular miRNAs. Our observation confirms a previous description that under the name of BN51, polr3d, has been found regulated by c-myc (Greasley et al., 2000). The period1 transcript (Figure 4B) was also dysregulated probably because this clock gene has been related to stress signaling. The clock transcript was not impacted by the delivery of miR-320-3p or its antagomiR. More experiments including CLOCK protein followed on later time points than 12H post-inoculation, are needed to clarify why our data are in contradiction with predicted binding site of miR-320-3p on clock transcript. In mice, mm-miR-320-3p is correlated with npas2 (Mo et al., 2017); however, we did not find any evolution of npas2 with the time of day nor nucleic acid delivery on rat pups (Figure 4F). Moreover, the physiological phenotype of every cell is maintained by highly collaborative program between epigenetic dynamics and metabolism which is further interconnected with other environmental cues provided by the circadian clock molecular machinery (Etchegaray and Mostoslavsky, 2016), but the circadian connection with a miRNA retaining an epigenetic property remains little explored. The miR-132 has been shown to couple the circadian clock to daily rhythms of neuronal plasticity and cognition (Aten et al., 2018). Potentially our system allows the transfer of any miRNA by an oral bolus with a high loading in gut tissues. More works are needed to confirm our data on the absence of miR-320-3p transfer in the brain by using selected miRNAs like miR-132 with well-described effect in the hippocampus by influencing neuronal morphology and memory.

The miRNA delivered to cells with appropriate carriers or expressed in cells using suitable vectors often trigger both intended sequence-specific silencing effects and unintended sequence-non-specific immune responses (Olejniczak et al., 2010). The transfection of 21 bp siRNAs to T98G cells resulted in the dose-dependent upregulation of STAT1 expression, as detected by Western blot and semi-quantitative RT–PCR analysis (Sledz et al., 2003). Our *in vivo* transfection concentrations were between 0.5 to 5 nM for 22 nucleotides miRNA (miR-320-3p with corresponding antagomiR). We did not find any up-regulation of stat1 (Figure 4D), only a non-significative trend at ZT-20H (*p* = 0.062). Likewise, no specific sequence for immunostimulation was found on miR-320-3p nor polr3d or hspb6. Our analysis was done on cells from the gastric wall and further works are needed to determine whether the stat-1 response is different according to cell lineage.

Endogenous levels of miR-21-5p or miR-29b-5p are unmodified by transfection of miR-320-3p in Human Embryonic Kidney cells (Kim et al., 2008). In our experiments, we did not find any effect on primiR-320-3p from 4 to 12 h post-transfection both in the stomach and liver (Figures 3E,F) nor on miR-21-5p (Supplementary Figure S7). However, loading of extracellular mature miRNA into recipient cells comes with a cost by impeding dynamic localization of miRNAs in nucleoli. As shown by *in situ* hybridization (Li et al., 2013) in human Hela cells, miRNAs are accumulating in different cellular organelles/compartment. In these cells, the miR-484 is given as specific of the nucleolar compartment. But, on our rat pups, miR-484 is detected both in cytoplasm and nucleus without any detectable accumulation in nucleoli. We have found that miR-320-3p is accumulating in nucleoli of gastric cells only at a low level (ZT-20H). More *in vivo* experiments designed in kinetic are needed to explore the dynamic of nucleole loading in transfected cells. If the existence of mm-miR-320-5p has been reported on RNA-Seq (Mo et al., 2017), we did not detect the -5p form in our experiments by q-PCR. We did not find any effect linked to competitive-endogenous-RNAs according to data on miR-320 measured in mouse hepatocytes cultured under various stressing conditions (Denzler et al., 2014).

We aim also to evaluate the possibility to impact the promoter *in vivo* by giving a very high concentration of miRNAs altering within hours, cellular physiology or phenotype. As a general rule derived from *in vitro* experiments (Mullokandov et al., 2012), we have used concentration for delivering at least 100 molecules in the gastric cell cytoplasm. With miR-320-3p supplemented group, the promoter polr3d was found in higher amount in immune complexes obtained with anti-H3K4me3 at ZT-20H (Figure 6A, *p* = 0.05). Kim et al. (2008) have shown that H3K27me3 and EZH2, a histone methyltransferase that mediates H3K27me3, were also enriched at the POLR3D promoter in HEK-293 cells with increased levels of miR-320. Benhamed et al., 2012 have reported that the senescent status of cells is promoting AGO-2 intranuclear localization and chromatin silencing. However, our data implement the antagonist role of H3K4me3 and H3K27me3 during the circadian cycle (Kim et al., 2013).

We have found a small hypoglycemic effect of an oral bolus of miR-320-3p (Supplementary Figure S6A), without modification of cholesterol, NEFA nor triglycerides levels (Supplementary Figures S6B–D). Our data are in line with the previous description of miR-320-3p properties (Beuzelin and Kaeffer, 2018) and in favor of the concept that miRNAs are playing a crucial role in canalization (Wu et al., 2009). However, we did not find any difference between sirt-1 nor on sirt-6 levels (Supplementary Figure S7). Having shown that high amount of miR-320-3p is associated in molecular complexes containing the polr3d promoter, the next step is to check whether *in vivo* inoculation of miRNAs targeting promoters, like miR-320-3p or miR-484, have a long-lasting effect on young and adult rats. In addition, the system DOSP/miRNA can be used as a positive control in experiments designed to prove a natural transfer of mature miRNA from food to consumer like a mother’s breast milk to the baby.

## Conclusion

In conclusion, extracellular miRNAs embedded in DOSP have immediate effects on RNAi and on nuclear chromatin complexes depending on a daily rhythm. In perinatal nutrition, an integrative view of the impact of extracellular miRNA on the physiology of laboratory rodents is deeply needed for assaying epigenetic manipulation under various environmental stresses.

In perspectives, the lack of knowledge on proper nutritional handling of preterm infants is a general health problem in the World (Harville et al., 2012) suspected to trigger the onset of a metabolic syndrome emerging later in older adult through nutritional programming (Lucas, 1994; Ma and Popkin, 2017). Targeting specific tissue to manipulate epigenetic profiling of gut cells during the perinatal period with miRNA embedded in nano-biomimetic vehicles devoid of inflammatory side effects can pave the way for the development of preventative strategies of metabolic syndrome induced in intensive care unit leading to diabetes (Beuzelin and Kaeffer, 2018).

## Data Availability

All datasets generated for this study are included in the manuscript and/or the Supplementary Files.

## Ethics Statement

Authorization of protocol was registered under #APAFIS-8406 by the National Ethics Committee and French Ministry of Agriculture.

## Author Contributions

DB and BK performed all the experiments. DB, BP, and BK wrote the manuscript.

## Conflict of Interest Statement

The authors declare that the research was conducted in the absence of any commercial or financial relationships that could be construed as a potential conflict of interest.

## Supplementary Material

FIGURE S1 Screening of aminoglycosides *ex vivo* and *in vitro*. Cytotoxicity of dioleyl-succinyl-paromomycin (DOSP), dioleyl-succinyl-serinyl-tobramycin (DOSST), dioleyl-succinyl-ribostamycin (DOSRI), and dioleyl-succinyl-serinyl-ribostamycin (DOSSRI) was assessed by resazurin on HCTT16 cells at 5, 24, or 48 h after transfection **(A)**. Levels of miR-320-3p delivered by DOSP (gray box) and DOSST (green) compare to empty-vector-control (pink (DOSP) or green (DOSST) borders) or non-transfected-control (blue border) in gastric primary culture of rat pups **(B)**. Ratios of miR-320-3p levels at specified time on ZT-0H **(C)** in gastric gland explants (white background) or exfoliated cells (blue background) at 24 and 48 h after transfection (*n* = 4 per group). In the box plots, a black line within the box marks the median. The boundary of the box closest to zero indicates the 25th percentile and the boundary of the box farthest from zero indicates the 75th percentile. Whiskers above and below the box indicate the 10th and 90th percentiles. ^*^*P* < 0.05 and ^∗∗^*P* < 0.001 compared to control. ^$$$^*P* < 0.001 DOSP/DOSST mir-320-3p compared to DOSP/DOSST empty or miR-320-3p alone.

FIGURE S2 Time series of an oral bolus of miR-320-3p, antagomiR-320-3p or control on the distribution of miR-320-3p in seven organs and exfoliated gastric cells. Levels of miR-320-3p in stomach **(A)**, stomach exfoliated cells **(B)**, small intestine **(C)**, plasma **(D)** or liver **(E)**, adipose tissue **(F)**, plexus choroid **(G)**, and cortex **(H)** at 4, 8, and 12 h after the bolus (*n* = 1–2 per group). At the bottom, black arrow-heads remind time of bolus (either at ZT-0H or ZT-12H) and Light bulb that light-on is taken as synchronizer (ZeitGeber), ZT-0H.

FIGURE S3 Evolution of gastric extracellular vesicles according to time after bolus. Sizing and counting of gastric extracellular vesicles were measured by Tunable Resistive Pulse Sensing (qNano) with a nanopore of 150 nm (NP150). We have analyzed protein fractions prepared by centrifuged gastric fluids of 12-day rat pups according to day (**A–D**; for control-bolus at 0, 4, 8, and 12 ZT-H, miR-320-3p-bolus at 4, 8, and 12 ZT-H, antagomiR-320-3p at 4, 8, and 12 ZT-H) or to night (**E–H**; for control-bolus at 12, 16, 20, and 24 ZT-H, miR-320-3p-bolus at 16, 20, and 24 ZT-H). Synchronizer time (ZeitGeber) is given by Light-on. Note the similarity of profiles in sizes and counts.

FIGURE S4 Detection of miR-320-3p in protein fraction of gastric fluids, 8 h after bolus. Note that no difference was found between groups. At the bottom, black arrow-heads remind time of bolus (either at ZT-0H or ZT-12H) and Light bulb that light-on is taken as synchronizer (ZeitGeber), ZT-0H. In the box plots, a black line within the box marks the median. The boundary of the box closest to zero indicates the 25th percentile and the boundary of the box farthest from zero indicates the 75th percentile. Whiskers above and below the box indicate the 10th and 90th percentiles.

FIGURE S5 Heterogeneity of miR-320-3p loading of nucleus and cytoplasm purified from gastric tissues at ZT-20H. Note that the group treated with miR-320-3p displayed a strickingly higher heterogeneity. Synchronizer time (ZeitGeber) is given by Light-on at ZT-0H. In the box plots, a black line within the box marks the median. The boundary of the box closest to zero indicates the 25th percentile and the boundary of the box farthest from zero indicates the 75th percentile. Whiskers above and below the box indicate the 10th and 90th percentiles.

FIGURE S6 Blood and plasma parameters according to ZT-8H or ZT-20H for rat pups receiving an oral bolus of miR-320-3p (blue), antagomiR-320-3p (magenta), or control (black). Blood glucose for experiments realized at light-on **(A)** or off **(B)**. Blood glucose **(C)**, Cholesterol **(D)**, Triglycerides **(E)**, and Non-Essential Fatty Acid (NEFA) **(F)** concentration of mir-320-3p (blue border), antagomiR-320-3p (magenta), or control (black box) groups. Data are mean ± sem, *n* = 5–6 rat pups per group. ^$^*P* < 0.05 and ^$$$^*P* < 0.001 ZT-8H compared to ZT-20H. At the bottom, black arrow-heads remind time of bolus (either at ZT-0H or ZT-12H) and light bulb that light-on is taken as synchronizer (ZeitGeber), ZT-0H. In the box plots, a black line within the box marks the median. The boundary of the box closest to zero indicates the 25th percentile and the boundary of the box farthest from zero indicates the 75th percentile. Whiskers above and below the box indicate the 10th and 90th percentiles.

FIGURE S7 Evaluation of miR-21-5p, primiR-21 in stomach and liver, sirt-1, sirt-6 and Cyclin D1 in stomach following miR-320-3p or antagomiR oral delivery. Levels of miR-21-5p in stomach **(A)** or liver **(C)** according to a bolus with miR-320-3p (blue box border), antagomiR-320-3p (magenta), and control (black). In parallel, the levels of primiR-21 in stomach **(B)** and liver **(D)** were determined. The mRNA levels of sirt-1 **(E)**, sirt-6 **(F)**, and cyclin-D1 **(G)** in stomach. In the box plots, a black line within the box marks the median. The boundary of the box closest to zero indicates the 25th percentile and the boundary of the box farthest from zero indicates the 75th percentile. Whiskers above and below the box indicate the 10th and 90th percentiles. We had 5–6 rat pups per group. ^$$^*P* < 0.01 ZT-8H compared to ZT-20H. At the bottom, black arrow-heads remind time of bolus (either at ZT-0H or ZT-12H) and light bulb that light-on is taken as synchronizer (ZeitGeber), ZT-0H.

FIGURE S8 Evaluation of c-myc **(A)**, period1 **(B)**, and stat1 **(C)** transcript expressions in liver. At the bottom, black arrow-heads remind time of bolus (either at ZT-0H or ZT-12H) and light bulb that light-on is taken as synchronizer (ZeitGeber), ZT-0H. In the box plots, a black line within the box marks the median. The boundary of the box closest to zero indicates the 25th percentile and the boundary of the box farthest from zero indicates the 75th percentile. Whiskers above and below the box indicate the 10th and 90th percentiles.

FIGURE S9 Western blot bands for POLR3D and HSPB6 comparatively to beta-ACTIN in stomach **(A)** and liver **(B)**. Note the difference in POLR3D expression at ZT-8H and ZT-20H for control groups in stomach **(A)** but not in liver **(B)**. For HSPB6 the expression at the difference of expression between ZT-8H and ZT-20H is seen for liver **(B)** but not for stomach **(A)**. At ZT-20H both for miR-320-3p and antagomiR groups, POLR3D expression is higher than for control.

FIGURE S10 Promoter polr3d detection in immune complexes obtained with anti-AGO1. At the bottom, black arrow-heads remind time of bolus (either at ZT-0H or ZT-12H) and light bulb that light-on is taken as synchronizer (ZeitGeber), ZT-0H. In the box plots, a black line within the box marks the median. The boundary of the box closest to zero indicates the 25th percentile and the boundary of the box farthest from zero indicates the 75th percentile. Whiskers above and below the box indicate the 10th and 90th percentiles.

TABLE S1 Raw data corresponding to the tracking of miR-320-3p in rat pup organs after loading at light on (ZT-8H) or off (ZT-20H). The file contains q-PCR data in Cq of miR-320-3p detection in stomach or liver of the experimental groups.

TABLE S2 Raw data corresponding to the evaluation of c-myc, circadian clock transcripts and stat-1, in stomach and liver, following miR-320-3p or antagomiR oral delivery. The file contains q-PCR data in Cq of miR-320-3p detection in stomach or liver of the experimental groups.

TABLE S3 Raw data corresponding to the expression of polr3d and hspb6, target genes, in stomach and liver, of miR-320-3p in rat pups receiving *per os*, miR-320-3p or antagomiR-loaded DOSP nanoparticles or control buffer. The file contains q-PCR data in Cq of miR-320-3p detection in stomach or liver of the experimental groups.

TABLE S4 Raw data corresponding to the difference between Chromatin-ImmunoPrecipitation done with anti-Trimethyl-histone-3-Lys4 or Lys-27 on gastric tissues of rat pups treated *per os*, with miR-320-3p or antagomiR-loaded DOSP nanoparticles or control buffer. The file contains q-PCR data in Cq of the experimental groups for ChIP experiment and detection of miR-320-3p in immune complexes.

TABLE S5 Raw data corresponding to the tracking of miR-320-3p in rat pup organs after loading at light on (ZT-8H) or off (ZT-20H) from 0, 4, 8, and 12 h. The file contains q-PCR data in Cq of miR-320-3p detection in stomach of the experimental groups.
