# Supplementary figures and images for: Oral Delivery of miRNA With Lipidic Aminoglycoside Derivatives in the Breastfed Rat
Source: Front Physiol. 2019 Aug 13;10:1037. doi: 10.3389/fphys.2019.01037 (PMC6700720; doi:10.3389/fphys.2019.01037)

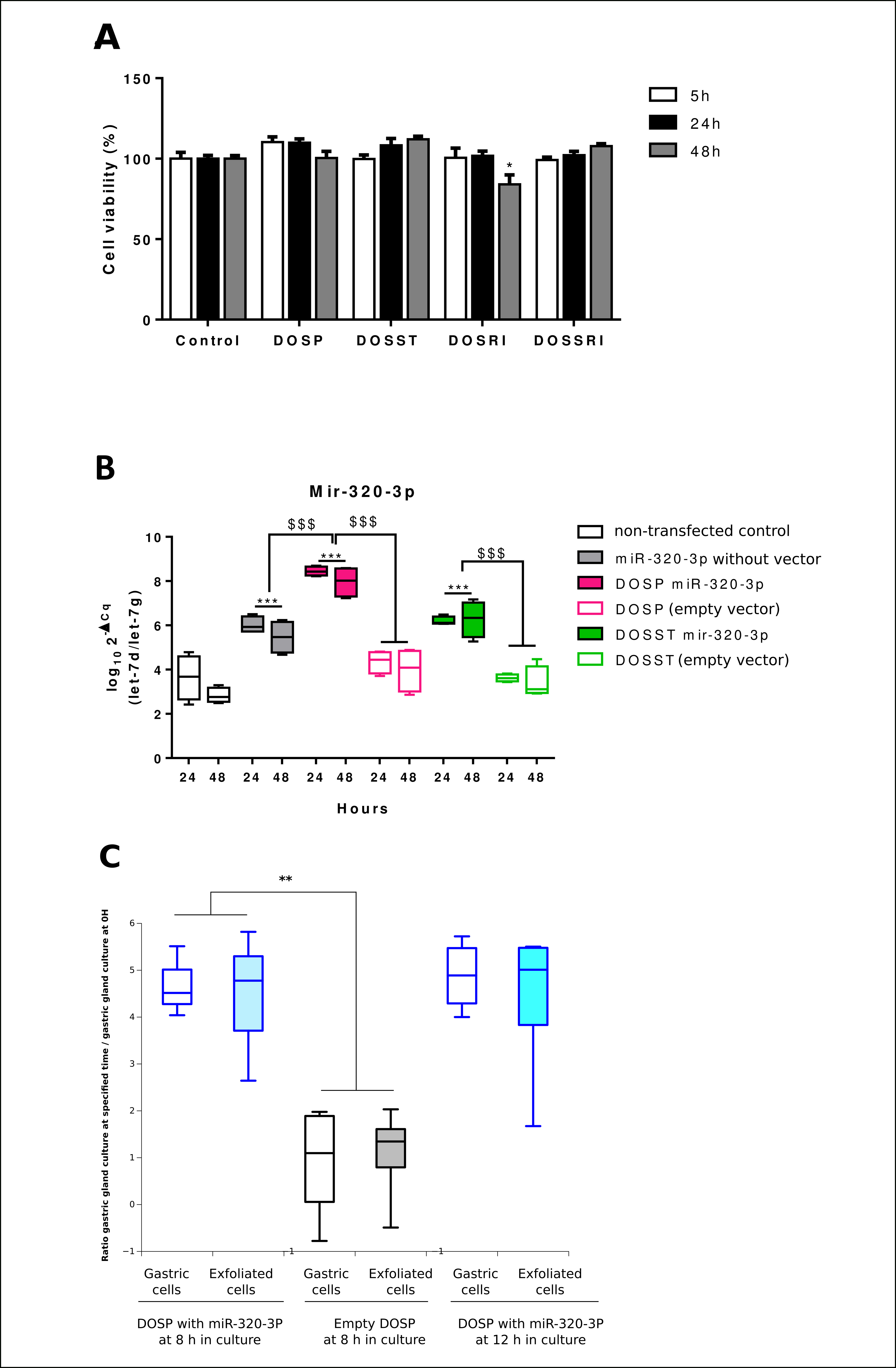

Supplement: FIGURE S1 — Screening of aminoglycosides ex vivo and in vitro. Cytotoxicity of dioleyl-succinyl-paromomycin (DOSP), dioleyl-succinyl-serinyl-tobramycin (DOSST), dioleyl-succinyl-ribostamycin (DOSRI), and dioleyl-succinyl-serinyl-ribostamycin (DOSSRI) was assessed by resazurin on HCTT16 cells at 5, 24, or 48 h after transfection (A). Levels of miR-320-3p delivered by DOSP (gray box) and DOSST (green) compare to empty-vector-control (pink (DOSP) or green (DOSST) borders) or non-transfected-control (blue border) in gastric primary culture of rat pups (B). Ratios of miR-320-3p levels at specified time on ZT-0H (C) in gastric gland explants (white background) or exfoliated cells (blue background) at 24 and 48 h after transfection (n = 4 per group). In the box plots, a black line within the box marks the median. The boundary of the box closest to zero indicates the 25th percentile and the boundary of the box farthest from zero indicates the 75th percentile. Whiskers above and below the box indicate the 10th and 90th percentiles. *P < 0.05 and ∗∗P < 0.001 compared to control. $$$P < 0.001 DOSP/DOSST mir-320-3p compared to DOSP/DOSST empty or miR-320-3p alone. [file Image_1.JPEG]

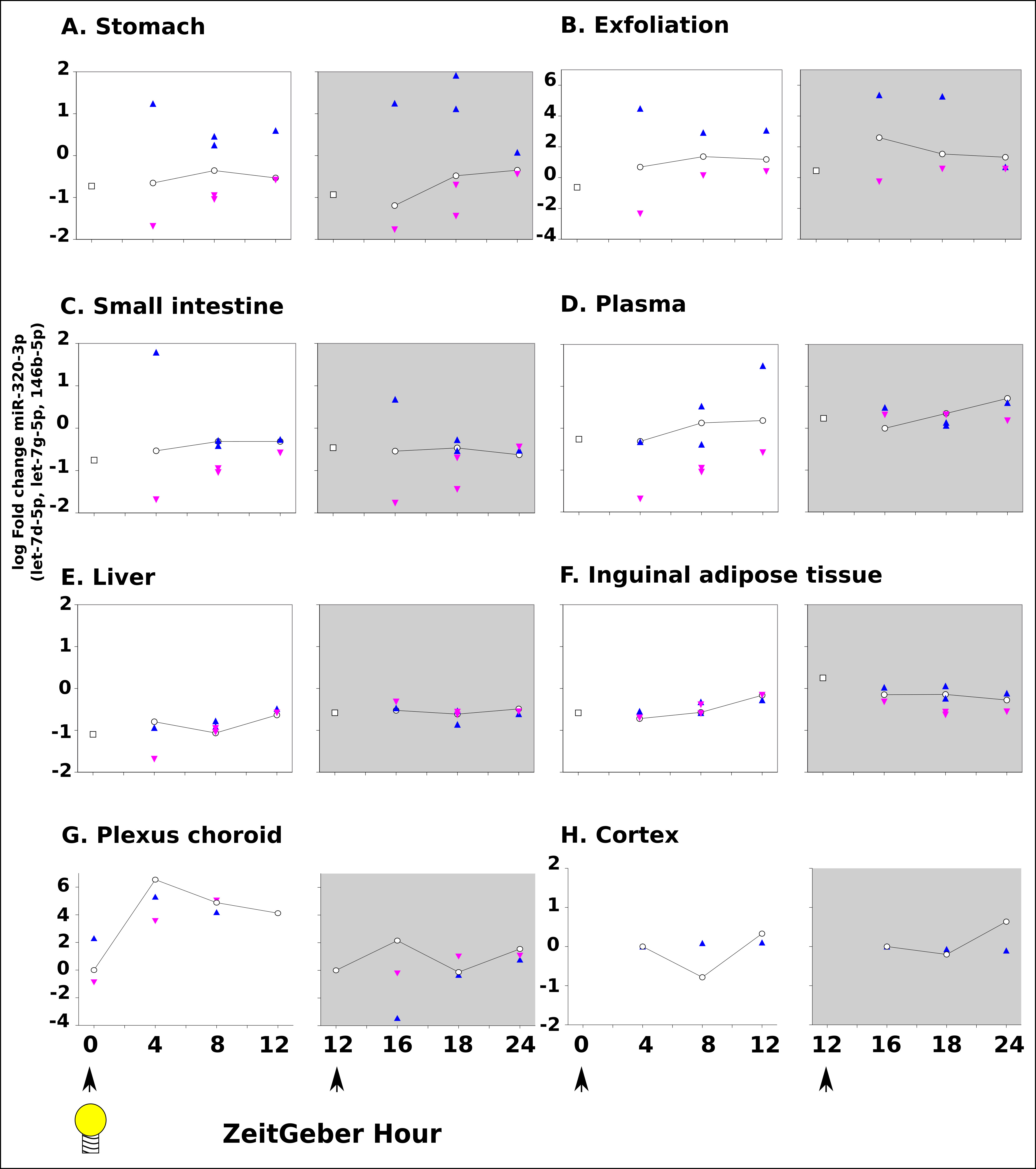

Supplement: FIGURE S2 — Time series of an oral bolus of miR-320-3p, antagomiR-320-3p or control on the distribution of miR-320-3p in seven organs and exfoliated gastric cells. Levels of miR-320-3p in stomach (A), stomach exfoliated cells (B), small intestine (C), plasma (D) or liver (E), adipose tissue (F), plexus choroid (G), and cortex (H) at 4, 8, and 12 h after the bolus (n = 1–2 per group). At the bottom, black arrow-heads remind time of bolus (either at ZT-0H or ZT-12H) and Light bulb that light-on is taken as synchronizer (ZeitGeber), ZT-0H. [file Image_2.JPEG]

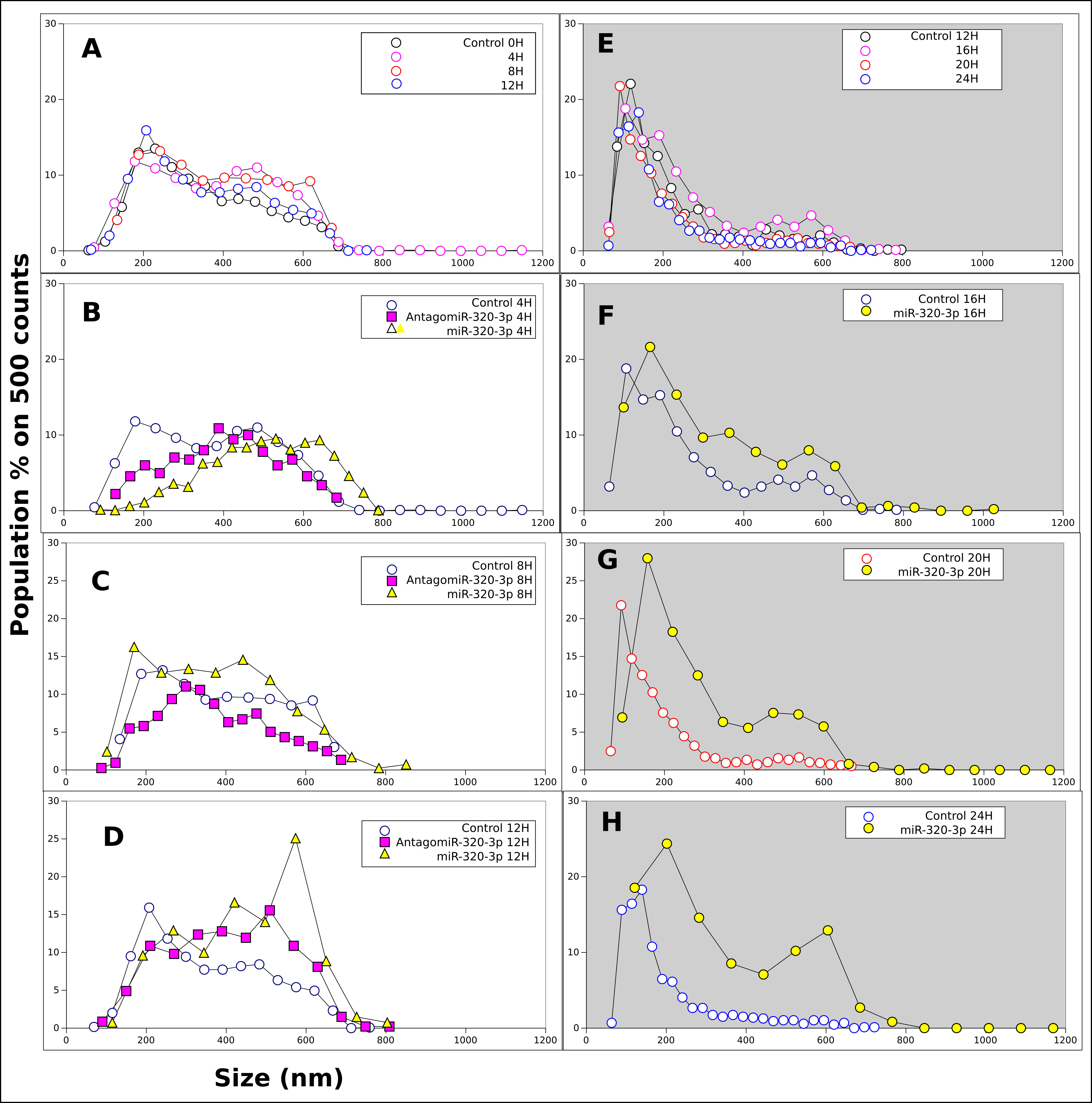

Supplement: FIGURE S3 — Evolution of gastric extracellular vesicles according to time after bolus. Sizing and counting of gastric extracellular vesicles were measured by Tunable Resistive Pulse Sensing (qNano) with a nanopore of 150 nm (NP150). We have analyzed protein fractions prepared by centrifuged gastric fluids of 12-day rat pups according to day (A–D; for control-bolus at 0, 4, 8, and 12 ZT-H, miR-320-3p-bolus at 4, 8, and 12 ZT-H, antagomiR-320-3p at 4, 8, and 12 ZT-H) or to night (E–H; for control-bolus at 12, 16, 20, and 24 ZT-H, miR-320-3p-bolus at 16, 20, and 24 ZT-H). Synchronizer time (ZeitGeber) is given by Light-on. Note the similarity of profiles in sizes and counts. [file Image_3.JPEG]

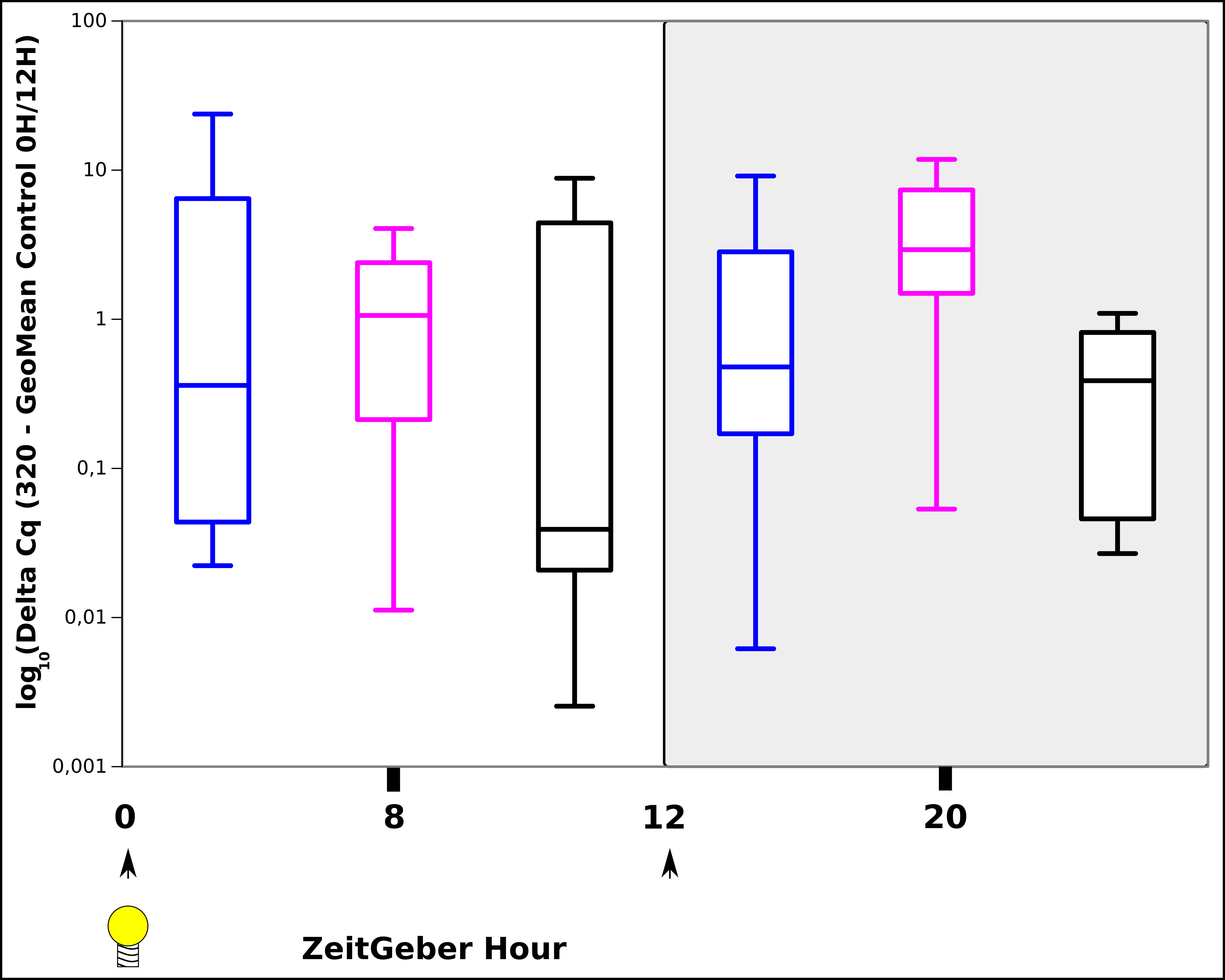

Supplement: FIGURE S4 — Detection of miR-320-3p in protein fraction of gastric fluids, 8 h after bolus. Note that no difference was found between groups. At the bottom, black arrow-heads remind time of bolus (either at ZT-0H or ZT-12H) and Light bulb that light-on is taken as synchronizer (ZeitGeber), ZT-0H. In the box plots, a black line within the box marks the median. The boundary of the box closest to zero indicates the 25th percentile and the boundary of the box farthest from zero indicates the 75th percentile. Whiskers above and below the box indicate the 10th and 90th percentiles. [file Image_4.JPEG]

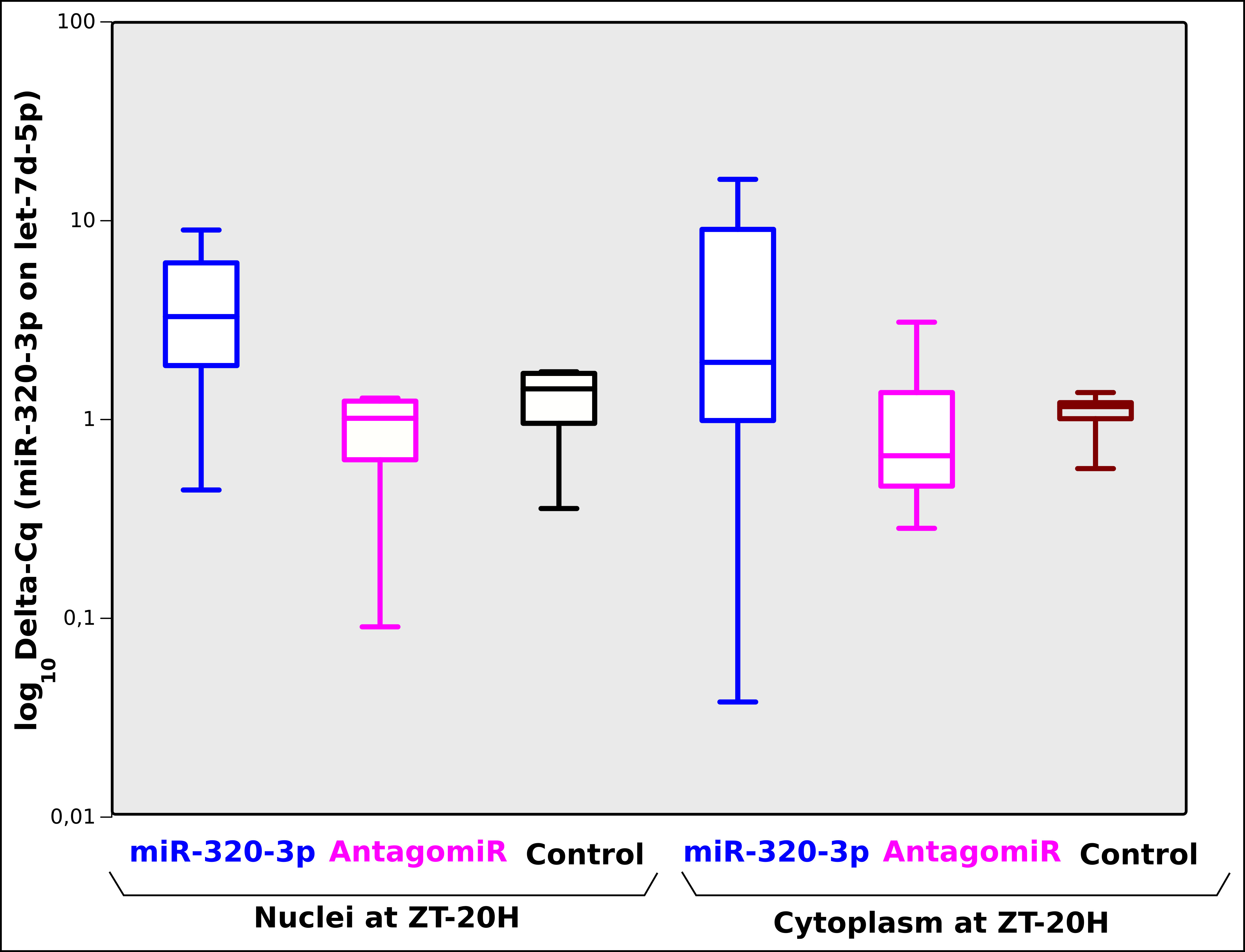

Supplement: FIGURE S5 — Heterogeneity of miR-320-3p loading of nucleus and cytoplasm purified from gastric tissues at ZT-20H. Note that the group treated with miR-320-3p displayed a strickingly higher heterogeneity. Synchronizer time (ZeitGeber) is given by Light-on at ZT-0H. In the box plots, a black line within the box marks the median. The boundary of the box closest to zero indicates the 25th percentile and the boundary of the box farthest from zero indicates the 75th percentile. Whiskers above and below the box indicate the 10th and 90th percentiles. [file Image_5.JPEG]

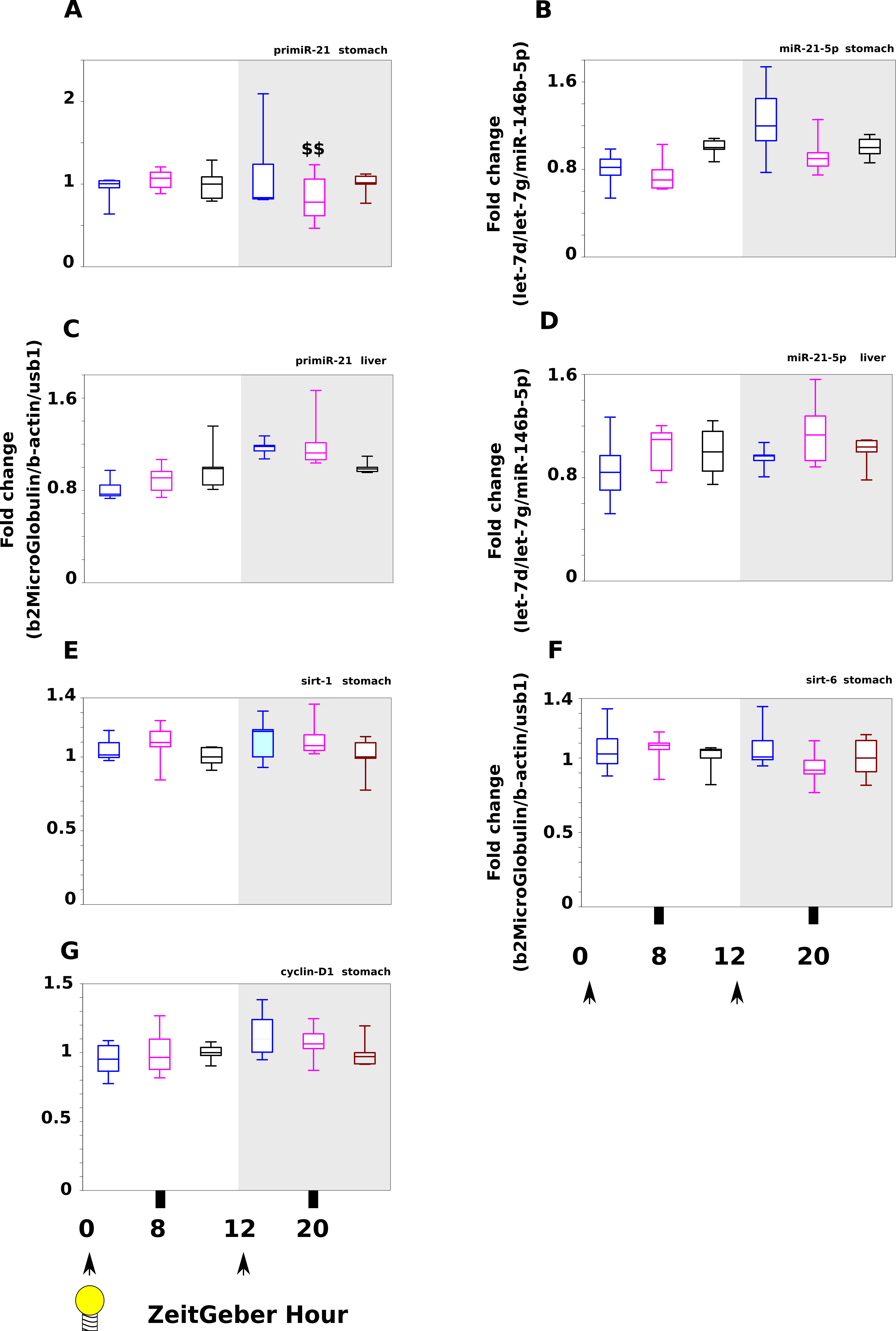

Supplement: FIGURE S7 — Evaluation of miR-21-5p, primiR-21 in stomach and liver, sirt-1, sirt-6 and Cyclin D1 in stomach following miR-320-3p or antagomiR oral delivery. Levels of miR-21-5p in stomach (A) or liver (C) according to a bolus with miR-320-3p (blue box border), antagomiR-320-3p (magenta), and control (black). In parallel, the levels of primiR-21 in stomach (B) and liver (D) were determined. The mRNA levels of sirt-1 (E), sirt-6 (F), and cyclin-D1 (G) in stomach. In the box plots, a black line within the box marks the median. The boundary of the box closest to zero indicates the 25th percentile and the boundary of the box farthest from zero indicates the 75th percentile. Whiskers above and below the box indicate the 10th and 90th percentiles. We had 5–6 rat pups per group. $$P < 0.01 ZT-8H compared to ZT-20H. At the bottom, black arrow-heads remind time of bolus (either at ZT-0H or ZT-12H) and light bulb that light-on is taken as synchronizer (ZeitGeber), ZT-0H. [file Image_7.JPEG]

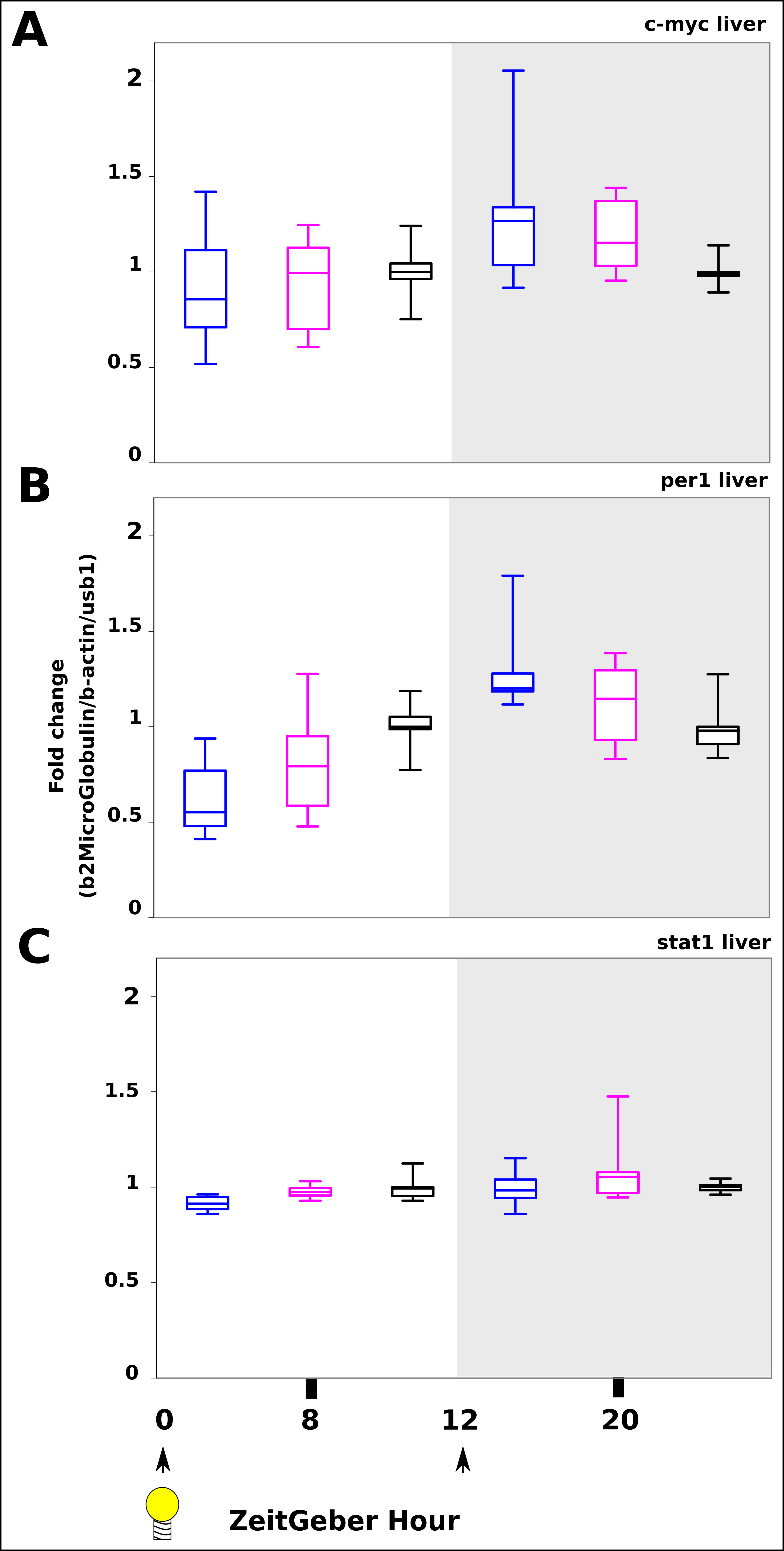

Supplement: FIGURE S8 — Evaluation of c-myc (A), period1 (B), and stat1 (C) transcript expressions in liver. At the bottom, black arrow-heads remind time of bolus (either at ZT-0H or ZT-12H) and light bulb that light-on is taken as synchronizer (ZeitGeber), ZT-0H. In the box plots, a black line within the box marks the median. The boundary of the box closest to zero indicates the 25th percentile and the boundary of the box farthest from zero indicates the 75th percentile. Whiskers above and below the box indicate the 10th and 90th percentiles. [file Image_8.JPEG]

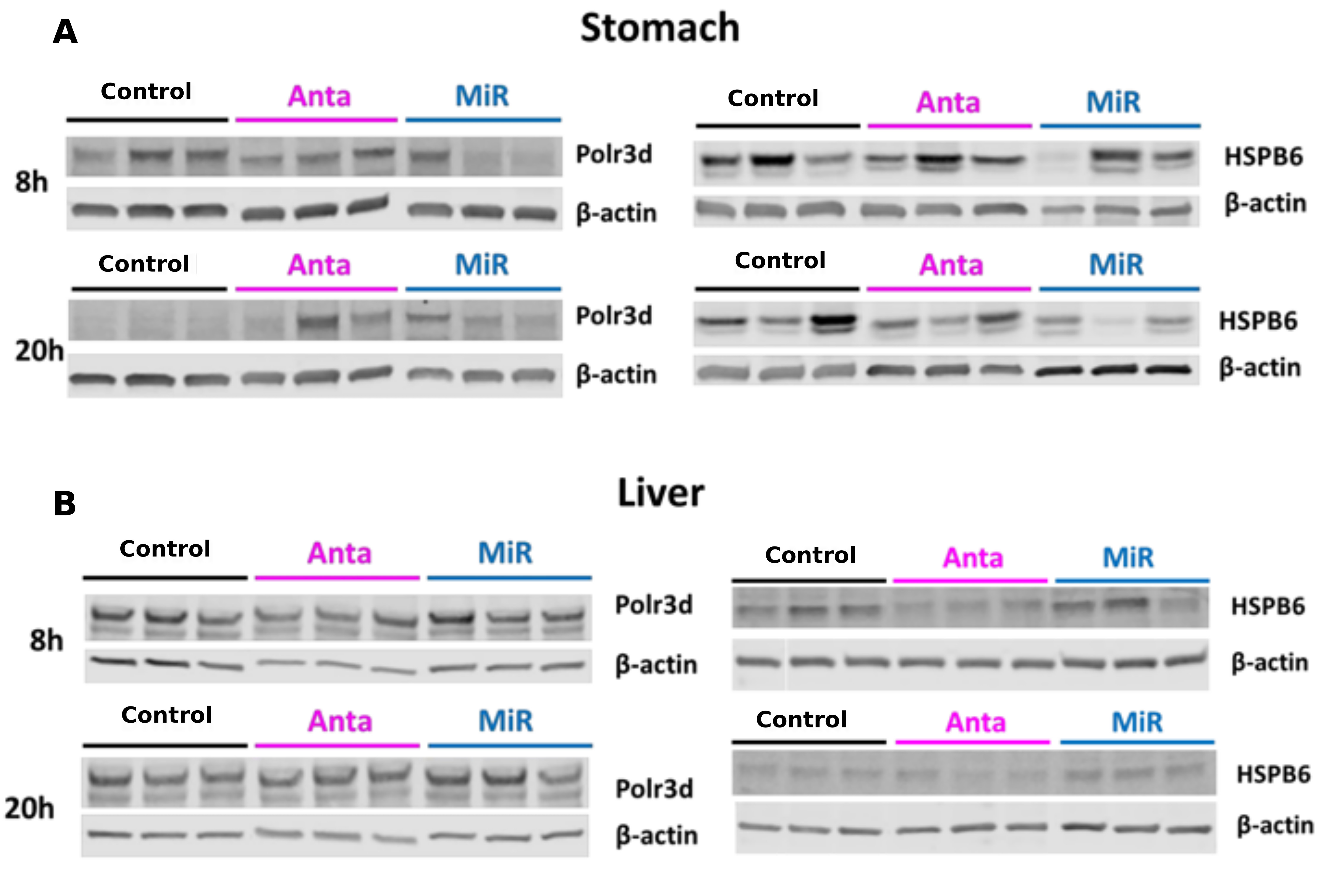

Supplement: FIGURE S9 — Western blot bands for POLR3D and HSPB6 comparatively to beta-ACTIN in stomach (A) and liver (B). Note the difference in POLR3D expression at ZT-8H and ZT-20H for control groups in stomach (A) but not in liver (B). For HSPB6 the expression at the difference of expression between ZT-8H and ZT-20H is seen for liver (B) but not for stomach (A). At ZT-20H both for miR-320-3p and antagomiR groups, POLR3D expression is higher than for control. [file Image_9.JPEG]

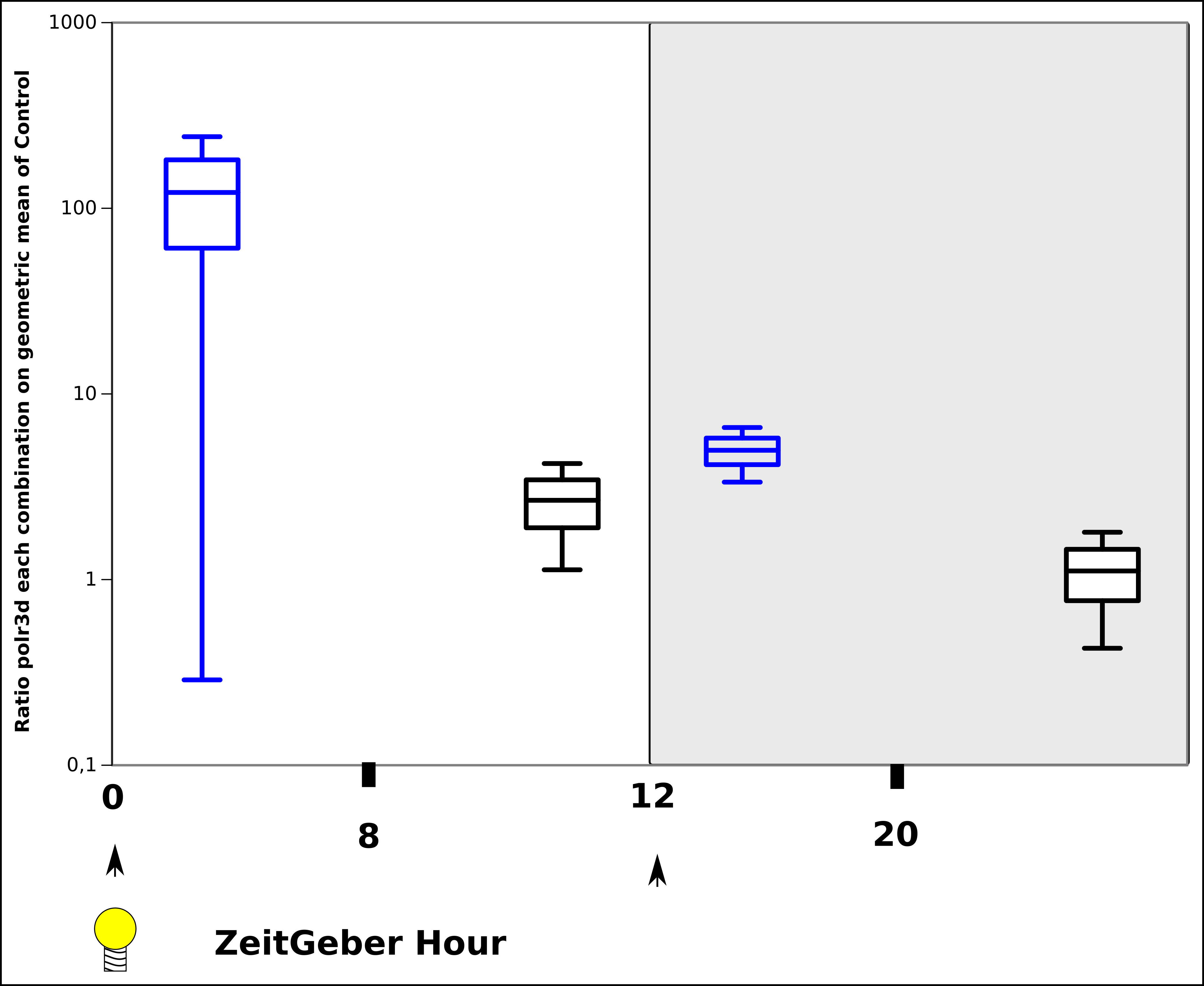

Supplement: FIGURE S10 — Promoter polr3d detection in immune complexes obtained with anti-AGO1. At the bottom, black arrow-heads remind time of bolus (either at ZT-0H or ZT-12H) and light bulb that light-on is taken as synchronizer (ZeitGeber), ZT-0H. In the box plots, a black line within the box marks the median. The boundary of the box closest to zero indicates the 25th percentile and the boundary of the box farthest from zero indicates the 75th percentile. Whiskers above and below the box indicate the 10th and 90th percentiles. [file Image_10.JPEG]
